# The Effects of Weaning Methods on Gut Microbiota Composition and Horse Physiology

**DOI:** 10.3389/fphys.2017.00535

**Published:** 2017-07-25

**Authors:** Núria Mach, Aline Foury, Sandra Kittelmann, Fabrice Reigner, Marco Moroldo, Maria Ballester, Diane Esquerré, Julie Rivière, Guillaume Sallé, Philippe Gérard, Marie-Pierre Moisan, Léa Lansade

**Affiliations:** ^1^UMR 1313, Institut National de la Recherche Agronomique, AgroParisTech, Université Paris-Saclay Jouy-en-Josas, France; ^2^UMR 1286, Institut National de la Recherche Agronomique, Université Bordeaux, Nutrition et Neurobiologie Intégrée Bordeaux, France; ^3^AgResearch Ltd, Grasslands Research Centre Palmerston North, New Zealand; ^4^UMR 1282, Institut National de la Recherche Agronomique, Infectiologie et Santé Publique Nouzilly, France; ^5^Departament de Genètica i Millora Animal, Institut de Recerca i Tecnologia Agroalimentàries, Torre Marimon Caldes de Montbui, Spain; ^6^UMR 444, Institut National de la Recherche Agronomique, Plateforme GET Castanet-Tolosan, France; ^7^UMR 1319, Institut National de la Recherche Agronomique, AgroParisTech, Université Paris-Saclay Jouy-en-Josas, France; ^8^PRC, Institut National de la Recherche Agronomique, Centre National de la Recherche Scientifique, IFCE, Université de Tours Nouzilly, France

**Keywords:** horse, gut microbiota, maternal separation, stress, weaning

## Abstract

Weaning has been described as one of the most stressful events in the life of horses. Given the importance of the interaction between the gut-brain axis and gut microbiota under stress, we evaluated (i) the effect of two different weaning methods on the composition of gut microbiota across time and (ii) how the shifts of gut microbiota composition after weaning affect the host. A total of 34 foals were randomly subjected to a progressive (P) or an abrupt (A) weaning method. In the P method, mares were separated from foals at progressively increasing intervals every day, starting from five min during the fourth week prior to weaning and ending with 6 h during the last week before weaning. In the A method, mares and foals were never separated prior to weaning (0 d). Different host phenotypes and gut microbiota composition were studied across 6 age strata (days −30, 0, 3, 5, 7, and 30 after weaning) by 16S rRNA gene sequencing. Results revealed that the beneficial species belonging to *Prevotella, Paraprevotella*, and *Ruminococcus* were more abundant in the A group prior to weaning compared to the P group, suggesting that the gut microbiota in the A cohort was better adapted to weaning. *Streptococcus*, on the other hand, showed the opposite pattern after weaning. Fungal loads, which are thought to increase the capacity for fermenting the complex polysaccharides from diet, were higher in P relative to A. Beyond the effects of weaning methods, maternal separation at weaning markedly shifted the composition of the gut microbiota in all foals, which fell into three distinct community types at 3 days post-weaning. Most genera in community type 2 (i.e., *Eubacterium, Coprococcus, Clostridium* XI, and *Blautia* spp.) were negatively correlated with salivary cortisol levels, but positively correlated with telomere length and N-butyrate production. Average daily gain was also greater in the foals harboring a community type 2 microbiota. Therefore, community type 2 is likely to confer better stress response adaptation following weaning. This study identified potential microbial biomarkers that could predict the likelihood for physiological adaptations to weaning in horses, although causality remains to be addressed.

## Introduction

Weaning is a stressful and complex process involving affective, physiological, nutritional, and cognitive-behavioral responses in an attempt to regain homeostasis (Lansade et al., 2004; Waran et al., 2008). In horses, weaning has been described as one of the most stressful events in life (Erber et al., 2012). Weaning results in an increased frequency of vocalizations and general motor activity during the first days, as well as altered feeding and sleeping patterns, irritability, anxiousness, aggressiveness and suspension of play for longer periods (Henry et al., 2012). Many foals also experience elevated glucocorticoid levels, as well as weight loss, performance decline after weaning and higher risk of infectious diseases (Moons et al., 2005; Waran et al., 2008; Bruschetta et al., 2017).

Stress activates the sympatho-adrenomedullary (SAM) and the hypothalamic-pituitary-adrenal (HPA) axes, which release catecholamines and glucocorticoids into the circulatory system (Furay et al., 2008). There is increasing evidence that the gastrointestinal tract responds to stress hormones by synthesizing cytokines, hormones, and neurotransmitters (Holzer and Farzi, 2014), which might modify microbiota diversity and increase pathogen colonization (Lyte et al., 2011). One of the ways by which stress hormones can promote pathogenic bacterial growth is by facilitating adherence to the gut wall and the induction of virulence factors (i.e., K99 pilus adhesin in *Escherichia coli*, Lyte et al., 2011). On the other hand, gut microbiota composition can regulate the stress response by means of the synthesis of hormones and neurotransmitters such as serotonin, as well as short chain fatty acids (SCFA) or secondary bile acids (Lyte and Ernst, 1993; Lyte et al., 1996; Asano et al., 2012).

Many questions remain about whether shifts in gut microbiota composition are a cause or a consequence of the stress conditions, and about how to turn this knowledge into treatments (Eisenstein, 2016). Despite the uncertainties concerning this causal link, there are findings from humans and animal models that lend weight to the hypothesis that the gut microbiota composition is critical to the development and function of an appropriate stress response (Sudo et al., 2004). For example, the transfer of gut microbiota from saline-treated non-obese diabetic mice to naive controls induced social deficits and lowered expression of myelin-associated genes in the prefrontal cortex of recipients (Gacias et al., 2016). On the other hand, a recent human study based on a multiple-stressor military training environment elucidated how stress modifies gut microbiota composition (Karl et al., 2017). Compared with controls, stressed individuals showed an increased level of microbial diversity, an increased abundance of members of the commensal microbiota that may become pathogenic under certain circumstances (which are referred to here as pathobionts, Browne et al., 2017) and a decreased abundance of the dominant beneficial species, such as members of the *Bacteroidaceae*, and *Lachnospiraceae* families (Karl et al., 2017).

Horses are monogastric and hindgut fermenters (Costa and Weese, 2012) and harbor an estimated 10^9^ microorganisms per gram of ingesta in the cecum alone (Mackie and Wilkins, 1988). The horse gut microbiota is composed of ~108 genera (Steelman et al., 2012; Venable et al., 2017) belonging to at least seven phyla (Costa et al., 2012, 2015; Shepherd et al., 2012; Weese et al., 2015). Bacterial populations differ greatly throughout the various compartments of the equine gastrointestinal tract (i.e., duodenum, jejunum, ileum and colon) due to differences in the gut pH, available energy sources, epithelial architecture of each region, oxygen levels, and physiological roles (Costa et al., 2015; Ericsson et al., 2016).

The gut microbiota promotes digestion and nutrient absorption for host energy production (Hsu et al., 2015) and provides folate (Sugahara et al., 2015), vitamin K_2_(Marley et al., 1986) and SCFA such as acetate, butyrate, and propionate (Argenzio and Hintz, 1972; Milinovich et al., 2008; Biddle et al., 2013; Nedjadi et al., 2014; Ericsson et al., 2016) which contribute to 60–70% of energy for horses (Al Jassim and Andrews, 2009). The gut microbiota also neutralizes drugs and carcinogens, modulates intestinal motility, protects the host from pathogens, and stimulates and matures the immune system and epithelial cells (reviewed by Nicholson et al., 2012). In horses, it remains unclear whether gut microbiota play a key role in modulating the physiological mechanisms involved in the weaning stress response, including hormone and neurotransmitter synthesis, modulation of oxidative stress, inflammatory response, gut motility and permeability or protection from pathogens. Therefore, we hypothesized that the progressive weaning in foals may enable to reduce the stress levels at weaning, as previously suggested (Erber et al., 2012; Bruschetta et al., 2017), leading to the maintenance and development of a healthy gut microbiota (i.e., increased abundance of beneficial taxa and decreased abundance of pathobionts), as well as host physiological variables. Consequently, our primary aim in this study was to explore the effect of the weaning method on gut microbiota composition in foals across time. Secondary, we sought to characterize how changes in the gut microbiota composition and function immediately after weaning affects host physiology.

## Materials and methods

### Animals, treatments, and sampling

Thirty-four Welsh foals (19 females and 15 males) were studied at the Val de Loire Centre (Nouzilly, France) of the National Institute for Agricultural Research (INRA). All animal procedures were conducted according to the guidelines for the care and use of experimental animals established by INRA (Ability for animal experimentation: A78-172, agreement for experimentation at Nouzilly: A-17661; protocol approved by a local ethics committee COMETHEA Poitou-Charentes with the permit number: CE2013-2).

At 5 months old (−119 d before weaning), the foals were housed together with their mares in a large pen with straw bedding. At 7 months old (−30 d before weaning), foals were selected carefully accounting for covariates such as gender, age, parity of the mare, and environmental influences (i.e., disease state, antimicrobials, co-habitation) and distributed into 17 boxes (3.52 × 4.68 × 4.26 m) in groups made up by two foals and two mares. Foals came from five different sires (from 2 to 7 foals per sire). Since it has been established previously that gut microbiota profiles might be shaped by host genetics (Lozupone et al., 2012), only one foal per sire and pen was selected in order to avoid close genetic relationship, which may cause difficulties to understand the individual variance underlying microbiota composition and would require the use of statistical models including random effects with a variance co-variance matrix dependent on the family structure of the foals. The “kinship2” R package was used to create the numerator relationship matrix, which estimates the genetic parameters and predicts breeding values between animals. Both pedigree tree and correlation structure matrix are depicted in Figure S1.

To assess the effect of weaning methods, foals were randomly allocated to one of two weaning methods: “progressive weaning group” (P, *N* = 18; 8 males, 10 females) and an “abrupt weaning group” (A, *N* = 16; 7 males, 9 females). The P group consisted of 18 mare-foal pairs, housed in nine loose boxes, each housing two mare/foal dyads. The A group consisted of 16 mare-foal pairs housed in eight loose boxes of two mare/foal dyads.

In the P group, mares and foals were separated daily by a steel-fencing panel (1.20 × 1.55 m), starting at 4 weeks before weaning (0 d). Separation lasted 15 min on the first day and this duration was progressively increased by 2 min per day during the first week, 5 min per day during the second week, 20 min per day during the third week and 30 min per day during the fourth week, until separation lasted 6 h per day during the last week. Separation started at the same time each day to minimize the effect of circadian rhythm. The steel-fencing panel used to separate mares and foals allowed visual, olfactory and tactile contacts, but no suckling. However, during the 5 days prior to weaning, doors replaced fencing panels. As a consequence, no visual contact was allowed between foals and mares.

In the abrupt weaning group, mares and foals were never separated prior to weaning (0 d). All the other parameters were exactly the same for the two weaning groups (i.e., quantity and type of cereal-based diet, amount of hay provided per day, amount of handling, and box dimension).

All 34 foals were weaned (definitively separated from their mares, 0 d) at 250 ± 16 days of age and 149.5 ± 31.27 kg of body weight. Mares were fitted with a halter and transported by truck to a familiar stable located 2 km away. This late weaning time was chosen because it closely mimics the way in which mares wean their foals in the wild. In natural conditions, the foal progressively stops suckling between 11 and 12 months, just a few weeks or days prior to arrival of the next foal (Apter and Householder, 1996). Although under domestic conditions weaning used to take place around 6 months (Nicol et al., 2005; Waran et al., 2008; Lepeule et al., 2009), it seems to be more and more common to apply late weaning at present (around 8 months).

At weaning, foals are often subjected to a number of stressors (i.e., change in diet, separation from their mares and littermates, new environment), which together might modify the microbiota composition. Since we wanted to study the effect that weaning has on gut microbiota, after weaning the foals were left in their own loose boxes (same environment) with the same littermate. Additionally, plain oats and soybeans were already provided 1-month prior to weaning to familiarize the young animals with solid food and reduce the nutritional stress at weaning (−30 d). Precisely, from day −30 prior to weaning, foals received a mixture of oats and soybeans (450 g and ~30 g of dry matter (DM)/day per subject, respectively) in selective feed troughs (not accessible to mares). The nutritional composition of the cereal-based diet is summarized in Table S1. After weaning, foals continued to be fed daily with the same mixture. Feed quantities were adjusted every week to the foal body weight to cover the nutritional requirements of growing foals. During the first weak post-weaning, foals received 490 g (DM) of a mixture based on oats and soybeans per day, whereas during the second week post-weaning foals received 990 g (DM) of this mixture per day. A total of 1.30 kg (DM) per day of this mixture was supplemented from week 3 until the end of the experiment. Each morning, each single animal was fed 8 kg of hay using a hay net. The chemical composition of the hay was as follows: dry matter (DM; 777.1 g/kg), crude protein (72.9 g/kg DM), crude fiber (313.7 g/kg DM), ether extract (16.9 g/kg DM), ash (56.7 g/ kg DM), neutral detergent fiber (632.3 g/kg DM), acid detergent fiber (338.9 g/kg DM), acid detergent lignin (38.1 g/kg DM), and non-fiber carbohydrates (221.2 g/kg DM). Foals had access to water and a block of salt *ad libitum*.

**Figure 1:**
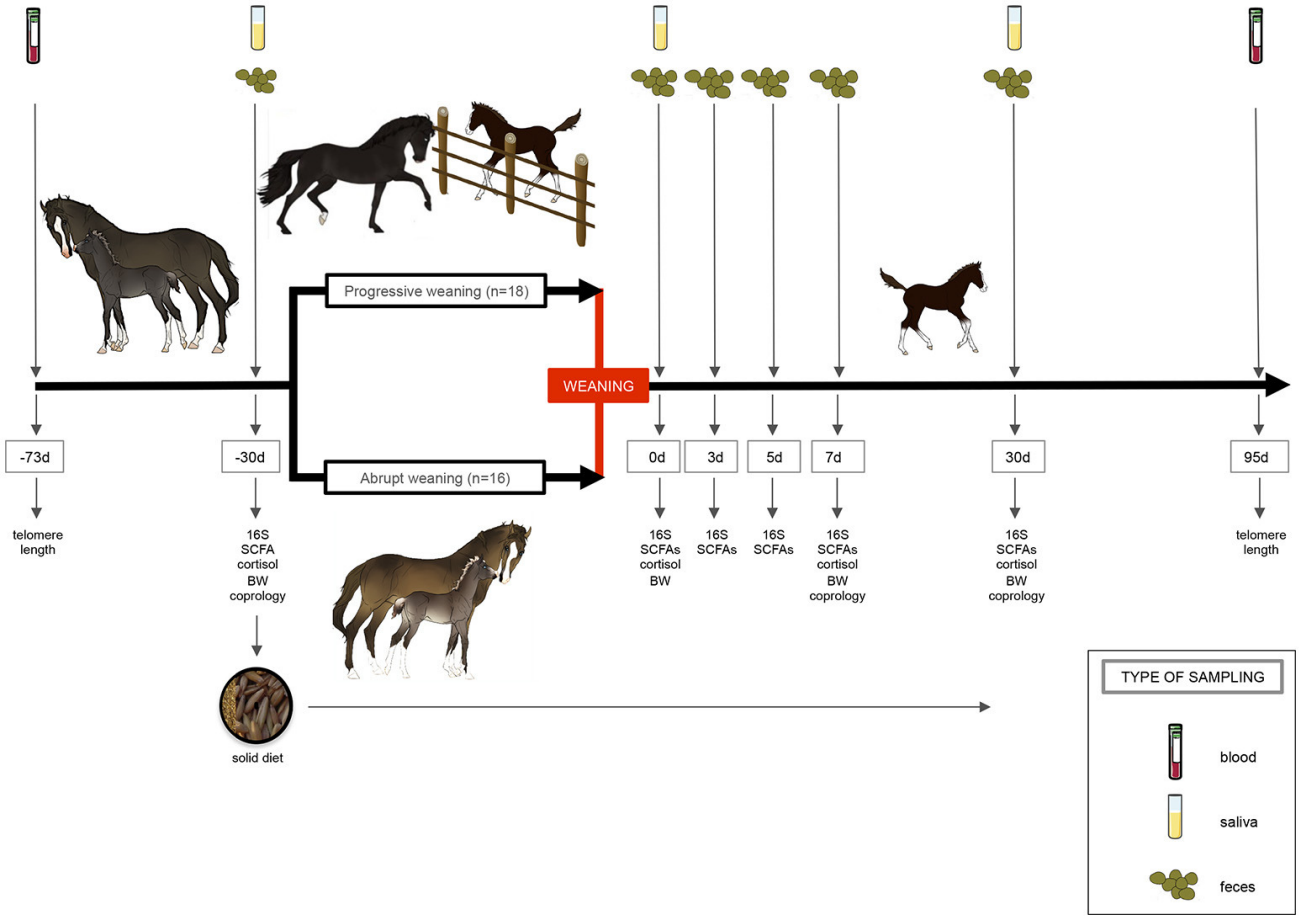
Experimental design and sampling. A total number of 34 foals were randomly allocated to one of two treatments: “progressive weaning” and an “abrupt weaning.” The progressive weaning started at day −30. In the progressive weaning, separation lasted 15 min on the first day and this duration was progressively increased until separation lasted 6 h per day the last week. In the abrupt weaning group, mares and foals were continuously housed together prior to weaning. Gut microbiota composition and short-chain fatty acids (SCFA), pH, as well as basal salivary cortisol, telomere length, parasite egg counts, and performance parameters were recorder through the experiment. A solid diet based on oats (90%) and soybean meals (10%) was provided 1 month prior to weaning (−30 d) to avoid the nutrition stress at weaning. This figure was created using images provided by Servier Medical Art (http://www.servier.com/Powerpoint-image-bank/).

Fresh fecal samples were obtained from all foals at −30, 0, 3, 5, 7, and 30 days post-weaning (Figure 1). While monitoring the foals from 6:00 to 8:00 a.m., one fecal sample from each animal was collected off the ground immediately after defecation. As described by Costa et al. (2012), ~10 g of feces were collected from the center of the fecal balls, avoiding collection of fecal material that was touching the ground. All fecal samples were snap-frozen in liquid nitrogen and stored at −80°C until use. At day 0 (0 d, weaning day), feces samples were taken before the separation between foals and mares. The pH was immediately determined after 10% fecal suspension (wt/vol) in saline solution (0.15 M NaCl solution).

SCFAs were measured in fecal samples as previously described (Lan et al., 2007).

Since cortisol rapidly diffuses into saliva and salivary cortisol reliably mirror changes in cortisol concentrations in blood plasma (Schmidt et al., 2010), basal salivary cortisol concentrations were sampled with Salivette ® Cortisol (SARSTEDT, France) in the morning (between 07:30 and 8:00 h) of days −30, 1, and 30 post-weaning. Cotton buds were centrifuged at 3000 g for 20 min at 4°C and the saliva was stored at −20°C until analysis. To minimize the stress response to handling during sampling (Lansade et al., 2004; Valenchon et al., 2017), foals were familiarized to the human presence and handling from the day of birth. As a consequence, the sampling procedure was well accepted by all foals and conducted by a single person without restraining the animals.

Additionally, blood samples for telomere length profiling were obtained from each animal at day −73 and 95 post-weaning using EDTA tubes (BD Vacutainer ®). After centrifugation, the packed blood cells were maintained at −20°C until DNA extraction.

For each foal, body weight and average daily gain were recorded until 30 days post-weaning. Each morning, daily concentrate and hay intake per pen was controlled from weaning (0 d) to 30 days post-weaning. Daily intake of hay per pen was measured and recorded as the total amount of fed provided each day minus the hay left, either in the hay net or on the floor of the stall. Similarly, the concentrate leftovers were measured and recorded daily from the feeders in each pen. However, because foals were housed in pairs within a pen, these data can only provide an estimation of the individually daily intake.

Since preliminary studies conducted in animals infected by gastrointestinal helminth reported significant changes in the composition of the gut microbiota (Walk et al., 2010; Li et al., 2012; Wu et al., 2012; Osborne et al., 2014), fresh fecal samples for parasitic coprology analysis (performed on 5 g of feces diluted in 70 mL of NaCl solution with density of 1.2; sensitivity of 50 eggs/g) were obtained at −30, 0, and 30 days post-weaning.

Infection, disease, and antibiotic treatment that may transiently alter the stability of the composition of the gut microbiota and thereby hamper the interpretation and comparison of community shifts between the weaning methods were controlled through the experiment. None of the individuals received antibiotic or parasite therapy during the sampling period. Foals were free of the principal equine infectious agents at the beginning and end of the study, and diarrhea was not detected in foals.

### Cortisol measurements

Cortisol concentration was measured in 50 μL of saliva using a luminescence immunoassay kit (Cortisol Saliva ELISA, IBL, Hamburg, Germany). The measurements were performed in a single assay. The intra-assay and inter-assay coefficients of variation were 4.7 and 10.6%, respectively. The assay sensitivity was 0.05 ng/ml.

### Telomere length measurement

Telomeres are repetitive nucleotide segments located at the ends of each mammalian chromosome that play a protective role during DNA transcription (Mathur et al., 2016) and can be shorten in proportion to the stress levels experienced early in life (Entringer et al., 2011). The telomere length measurement assay was adapted from the method described by Cawthon (2002). For each DNA sample, the ratio between telomere repeat copy number and the single copy gene copy number was calculated. The ratio was proportional to the average telomere length.

The forward primer for the telomere PCR was Tel1F [5′-CGGTTTGTTT GGGTTTGGGTTTGGGTTTGGGTTTGGGTT-3′] and the reverse primer was TelR [5′-GGCTTGCCTTACCCTTACCCTTACCCTTACCCTTACCCT-3′]. These specific forward and reverse primers were used at a final concentration of 300 and 900 nM, respectively. The single-copy gene was the interferon-γ (IFGM). The forward PCR primer for the IFGM was IFGMF [5′-ACGCAAGAAACCCAGATGTAGG-3′], and the reverse primer was IFGMR [5′-AGATATCCAGGAAAAGAGGCCC-3′]. These specific reverse and forward primers were used at a final concentration of 700 nM. All PCR primers were designed using ABI Primer Express software (PE Applied Biosystems, Courtaboeuf, France). The gDNA samples (2 μL of gDNA 10 ng/μL) were mixed with 1 × SYBR Green Master Mix (Roche, Mannhein, Germany) and 3 μL of the specific reverse and forward primers in a final volume of 10 μL. Tubes containing 20–10–5–2.5–1.2–0.6–0.3 ng/μL of a reference horse pooled DNA were included in each PCR run as a standard curve. The same standard curve was used for all PCR runs to evaluate the PCR efficiency of telomere and IFGM amplification. All PCRs were carried out on a Roche Lightcycler 480 real-time PCR machine with 384-tube capacity (Roche Diagnostics Corporation, Indianapolis, IN). Cycling conditions were 95°C for 10 min, then 40 cycles of 95°C for 10 s and 60°C for 6 s and 72°C for 10 s. For each sample and each gene, qPCR runs were performed in triplicate (in accordance with the manufacturer's protocol). In order to quantify and normalize the expression data, we used the ΔΔCt method. Values reported correspond to ΔΔCt between Ct value of telomeric region (T) amplification and IFGM (S) amplification for each sample relative to a control sample, here the value at day −73 for each weaning method. T/S value was calculated with GenEx software (GenEx 6.1, bioMCC, Germany). We used the amplification efficiency calculated by each standard curve on each plate.

### Ingestion behavioral observation

To evaluate the patterns of concentrate ingestion after weaning, we performed 18 observations per animal per day and we recorded if the foal ate its food or not at each time of observation. These observations were made each day from 0 d (i.e.; the day of weaning) to 3 days post-weaning. The observations were equally distributed all over the day (from 8.30 a.m. to 4.30 p.m.).

### Microorganisms DNA extraction from feces samples

Total DNA was extracted from aliquots of frozen fecal samples (200 mg; 204 samples at different age strata from 34 foals), using E.Z.N.A.® Stool DNA Kit (Omega Bio-Tek, Norcross, Georgia, USA). The DNA extraction protocol was carried out according to the manufacturer's instructions (Omega- Bio-Tek, Norcross, Georgia, USA).

### V3–V4 16S rRNA gene amplification

The V3-V4 hyper-variable regions of the 16S rDNA gene were amplified with two rounds of PCR using the forward primer (5′-CTTTCCCTACACGACGCTCTTCCGATCTACGGRAGGCAGCAG-3′) and the reverse primer (5′-GGAGTTCAGACGTGTGCTCTTCCGATCTTACCAGGGTATCTACT-3′) modified in order to include Illumina adapters and barcode sequences which allow for directional sequencing. The first round of amplification was performed in triplicate in a total volume of 50 μL containing 10 ng of DNA, 2.5 units of a DNA-free Taq DNA Polymerase and 10X Taq DNA polymerase buffer (MTP Taq DNA Polymerase, Sigma). Subsequently, 10 nmol of dNTP mixture (Euromedex, Souffelweyersheim, France), 20 μmol of each primer (Sigma, Lezennes, France) and Nuclease-free water (Ambion, Thermo Fisher Scientific, Waltham, USA) were added. Ultrapure Taq DNA polymerase, ultrapure reagents, and plastic were selected in order to be DNA-free. The thermal cycle consisted of an initial denaturation step (1 min at 94°C), followed by 30 cycles of denaturation (1 min at 94°C), annealing (1 min at 65°C) and 1 min of extension at 72°C. The final extension step was performed for 10 min at 72°C. Amplicons were then purified using magnetic beads (Clean PCR system, CleanNA, Alphen an den Rijn, The Netherlands) as follows: beads/PCR reactional volume ratio of 0.8X and final elution volume of 32 μL using Elution Buffer EB (Qiagen). The concentrations of the purified amplicons were checked using a NanoDrop 8000 spectrophotometer (Thermo Fisher Scientific, Waltham, USA).

Sample multiplexing was performed thanks to 6 bp unique indexes, which were added during the second PCR step at the same time as the second part of the P5/P7 adapters used for the sequencing step on the Illumina MiSeq flow cells with the forward primer (5′-AATGATACGGCGACCACCGAGATCTACACTCTTTCCCTACACGAC-3′) and reverse primer (5′-CAAGCAGAAGACGGCATACGAGATNNNNNNGTGACTGGAGTTCAGACGTGT-3′).

This second PCR step was performed using 10 ng of purified amplicons from the first PCR and adding 2.5 units of a DNA-free Taq DNA Polymerase and 10X MTP TaqDNA polymerase buffer (Sigma). The buffer was complemented with 10 mM of dNTP mixture (Euromedex), 20 mM of each primer (Eurogentec, HPLC grade) and Nuclease-free water (Ambion, Life Technologies) up to a final volume of 50 μL. The PCR reaction was carried out as follows: an initial denaturation step (94°C for 1 min), 12 cycles of amplification (94°C for 1 min, 65°C for 1 min and 72°C for 1 min) and a final extension step at 72°C for 10 min. Amplicons were purified as described for the first PCR round. The concentration of the purified amplicons was measured using Nanodrop 8000 spectrophotometer (Thermo Scientific) and the quality of a set of amplicons (12 samples per sequencing run) was checked using DNA 7500 chips onto a Bioanalyzer 2100 (Agilent Technologies, Santa Clara, CA, USA). All libraries were pooled at equimolar concentration in order to generate equivalent number of raw reads with each library. The final pool had a diluted concentration of 5 nM to 20 nM and was used for sequencing. Amplicon libraries were mixed with 15% PhiX control according to the Illumina's protocol. Details on sequencing, PhiX control and FastQ files generation are specified elsewhere (Lluch et al., 2015). For this study, one sequencing run was performed using MiSeq 500 cycle reagent kit v2 (2 × 250 output; Illumina, USA).

### Sequencing data preprocessing

Sequences were processed using the version 1.8.0 of the Quantitative Insights Into Microbial Ecology (QIIME) pipeline (Caporaso et al., 2010) and by choosing the open-reference operational taxonomic units (OTU) calling approach (Rideout et al., 2014).

First, forward and reverse paired-end sequence reads were collapsed into a single continuous sequence according to the “fastq-join” option of the “join_paired_ends.py” command in QIIME. Reads were joined using fastq-join with an allowed maximum difference within overlap region of 8%, a minimum overlap setting of 6 bp and a maximum overlap setting of 60 bp. The reads that did not overlap (~20% of the total; Table S2) were removed from the analysis. The retained sequences were then quality filtered. De-multiplexing, primer removal and quality filtering processes were performed using the “split_libraries”_fastq.py command in QIIME (Navas-Molina et al., 2013). We applied a default base call Phred threshold of 20, allowing maximum three low-quality base calls before truncating a read, including only reads with >75% consecutive high-quality base calls, and excluding reads with ambiguous (N) base calls (Navas-Molina et al., 2013). After the quality-filtering step, an average of 27,280 sequences per sample were obtained, with a mean length of 441 ± 15 bp.

Subsequently, the sequences were clustered into OTUs against the GreenGenes database (release 2013-08: gg_13_8_otus; DeSantis et al., 2006) by using the uclust (Edgar, 2010) method at a 97% similarity cutoff. The filtering of chimeric OTUs was performed by using Usearch (Edgar et al., 2011) against the GreenGenes reference alignment (DeSantis et al., 2006). A phylogenic tree was generated from the filtered alignment using FastTree (Price et al., 2010). Singletons were discarded from the dataset to minimize the effect of spurious, low abundance sequences using the “filter_otus_from_otu_table.py” script. To confirm the annotation, the resulting OTU representative sequences were then searched against the Ribosomal Database Project naïve Bayesian classifier (RDP 10 database, version 6, Cole et al., 2009) database, using the online program SEQMATCH (http://rdp.cme.msu.edu/seqmatch/seqmatch_intro.jsp). The sequences that were not assigned to the Bacteria kingdom were filtered out for the rest of the analysis. Finally, consensus taxonomy was provided for each OTU based on the taxonomic assignment of individual reads using GreenGenes and RDP. Using OTU abundance and the corresponding taxonomic classifications, feature abundance matrices were calculated at different taxonomic levels, representing OTUs and taxa abundance per sample. The “Phyloseq” R package (McMurdie and Holmes, 2013) was used for the detailed downstream analysis on abundance matrix. OTU counts per sample and OTU taxonomical assignments are available in Table S2.

The 16S rRNA amplicon gene sequences described in the study were deposited into the NCBI database (http://www.ncbi.nlm.nih.gov/). under GenBank accession numbers KY662487 to KY670589. The bioproject described in this paper belongs to the BioProject PRJNA375964. The corresponding BioSamples accession numbers were SAMN06348792 to SAMN06348995.

Species richness (Observed, Chao1; Chao, 1984) and α-diversity measurements (Shannon, 1997) were calculated using the “Phyloseq” R package (McMurdie and Holmes, 2013). Shannon's diversity index is a composite measure of richness (number of OTU present) and evenness (relative abundance of OTU). The nonparametric Wilcoxon rank-sum test was used to compare the species richness and α-diversity measurements between weaning methods.

Two different normalization methods were tested: (1) relative abundance normalization, which divides raw counts from a particular sample by the total number of reads in each sample; (2) the Cumulative-sum scaling (CSS) method from metagenomeSeq (Paulson et al., 2013), which divides raw counts by the cumulative sum of counts up to a percentile determined using a data-driven approach. This process generates an appropriate percentile for normalization for the data set, which can then be used for normalization before the application of Gaussian mixed models, which are used for inference in the metagenomeSeq pipeline.

To estimate β-diversity, weighted UniFrac distances were calculated from the OTU and genera abundance tables, and used in principal coordinates analysis (PCoA), non-parametric multidimensional scaling (NMDS), and sparse least squares discriminant analysis (sPLS-DA) with the “Phyloseq” R package. In addition to multivariate analysis, we used the analysis of similarities (ANOSIM) to test for intragroup dispersion. As specified by Poff et al. (2007), ANOSIM is a permutation-based test where the null hypothesis states that within-group distances are not significantly smaller than between-group distances. The test statistic (*R*) can range from 1 to −1, with a value of 1 indicating that all samples within groups are more similar to each other than to any other samples from different groups. *R* is ≈0 when the null hypothesis is true, that distances within and between groups are the same on average.

The Wilcoxon rank-sum test with Benjamini–Hochberg multiple test correction was used to determine the differentially abundant OTUs, families, and genera between groups. A *q* < 0.05 was considered significant.

### Functional metagenomic predictions

The functional prediction for the 16S rRNA marker gene sequences was done using PICRUSt (Langille et al., 2013). After excluding the OTU unknown from the GreenGenes reference database and normalizing by 16S rRNA gene copy number, functional metagenomes for each sample were predicted from the Kyoto Encyclopedia of Genes and Genomes (KEGG) catalog and collapsed to a specified KEGG level. We used Wilcoxon rank-sum test with Benjamini–Hochberg multiple test correction to evaluate pathway-level enrichments between groups. A *q* < 0.05 was considered as significant.

### Network inference at the genus level

Networks at the genus level were inferred at different time points. In order to prevent the compositional effects bias typical of the classical correlations methods (Aitchison, 1982; Pawlowsky-Glahn and Buccianti, 2011), we calculated the correlations among OTUs or genera using the REBECCA method (Ban et al., 2015), which identifies significant co-occurrence patterns by finding sparse solutions to a system with a deficient rank. This method constructs the system using log ratios of count data and solves the system using the l_1_-norm shrinkage method. In the network, every node represents one genus, whereas every edge connecting two nodes represents a significant interaction. We used the “iGraph” R package to visualize the network. As defined by Ramayo-Caldas et al. (2016), in the network, only those genera with sparse correlation ≥|0.15| were retained. Strong and significant correlation between nodes (*r* ≥ |0.60|) were represented with larger edge width.

### Effect of weaning methods on microbiota functions and host physiological parameters across time

The SCFAs, pH, the loads of fungi, protozoa and total bacteria in feces, as well as salivary cortisol, telomere length and performance phenotypes were analyzed using a mixed-effects analysis of the variance (ANOVA) model with repeated measures. The model included weaning method, time, and their interaction as fixed effects, and pen as random effects. To the extent that individual measurements on animals were not possible (i.e., intake of hay and concentrate), the pen was considered the experimental unit for the statistical model.

### Community type clustering across time

The intra- and inter-individual variations in the gut microbiota composition across time were studied based on the conceptual framework of enterotypes, or more generically, community types (Arumugam et al., 2011). According to this framework, the samples are clustered into bins based on their taxonomic similarity (Ding and Schloss, 2014). Briefly, we used the R Script available at: http://enterotype.embl.de/enterotypes.html; Arumugam et al., 2011) using the genera abundance of each individual (*n* = 34) in each time point (*n* = 6). Optimal number of communities' types in each time point was determined by the Calinski-Harabasz index. Species richness differences were calculated between communities type using the “Phyloseq” R package. The non-parametric Wilcoxon rank-sum test with Benjamini–Hochberg multiple test correction was used to compare the Chao1 index and the abundance of genera between gut community types in each time point.

### Analysis of the interaction between microbiota composition and host phenotypes

In order to find the possible associations between the gut community types and the host phenotypes (i.e., gender, age, salivary cortisol concentrations, telomere length, SCFAs, parasite egg counts, performance, and genetic background), we applied two different statistical approaches, namely: (1) mixed-effects ANOVA or Wilcoxon rank-sum tests conducted for continuous variables depending on the normality of the distribution of the input data to delineate whether there was a significant difference between the average values of phenotype traits for the different communities types, using a significance level of *p* < 0.05. The mixed-effects ANOVA model was also conducted to assess whether there was a significant difference between weaning method and pen for the different gut communities types; (2) Chi-square test with a threshold of *p* < 0.05 for discrete variables.

Next, the non-parametric Spearman rank correlation was performed to link genera relative abundances directly to phenotype traits for each time point. The non-parametric Spearman rank correlation was calculated between microbiota and phenotypic traits using the “corrplot” R package. The results were further justified by empirical permutation test. A two-sided *p* < 0.05 was considered significant.

### Real-time quantitative PCR (qPCR) analysis of bacterial, fungal and protozoan loads

Loads of protozoa, anaerobic fungi and bacteria in fecal samples were quantified using a QuantStudio 12K Flex real-time instrument (Thermo Fisher Scientific, Waltham, USA). Primers for real-time amplification of ciliates, anaerobic fungi, and bacteria have already been described (Kittelmann and Janssen, 2011; Kittelmann et al., 2012; Table S3) and have been purchased from Eurofins Genomics (Ebersberg, Germany).

Amplified fragments of the target genes were used and diluted 10-fold in series to produce seven standards, ranging from 2.25 × 10^7^ to 2.25 × 10^13^ copies per μg of DNA for bacteria and protozoa and ranging from 3.70 × 10^6^ to 3.70 × 10^12^ copies per μg of DNA for ciliates and fungi. Each reaction contained, in a final volume of 20 μL, 10 μL of Sybergreen Mix (Power SYBR Green PCR Master Mix, ThermoFisher, Ullkirch-Graffenstaden, France), 0.6 μM of each primer to final concentration of 300 mM, and 2 μL of standard or DNA template at 0.5 ng/μL. The primer concentration of anaerobic fungi was 200 mM and 15 mM for ciliate protozoa. The DNA template was 0.5 ng/μL. In all cases, the thermal protocol for qPCR amplification and detection included an initial step of denaturation of 10 min (95°C), followed by 40 amplification cycles [15 s at 95°C; 60 s at 60°C]. After each run, melting curves between 60 and 95°C were evaluated to confirm the absence of unspecific signals. For each sample and each gene, qPCR runs were performed in triplicate. The standard curve obtained the reference genomic fragment was used to calculate the number of copies of bacteria, protozoa, or anaerobic fungi in feces. Taking into account the molecular mass of nucleotides and fragment length, we calculated the copy number as follows: mass in Daltons (g/mol) = (size of double-stranded [ds] product in base pairs [bp]) (330 Da × 2 nucleotides [nt]/bp) (Whelan et al., 2003). Wilcoxon rank-sum tests were calculated for all possible group combinations and corrected for multiple testing using Benjamini–Hochberg false discovery rate (*q*-value). A *q* < 0.05 was considered significant.

## Results

**Figure 2:**
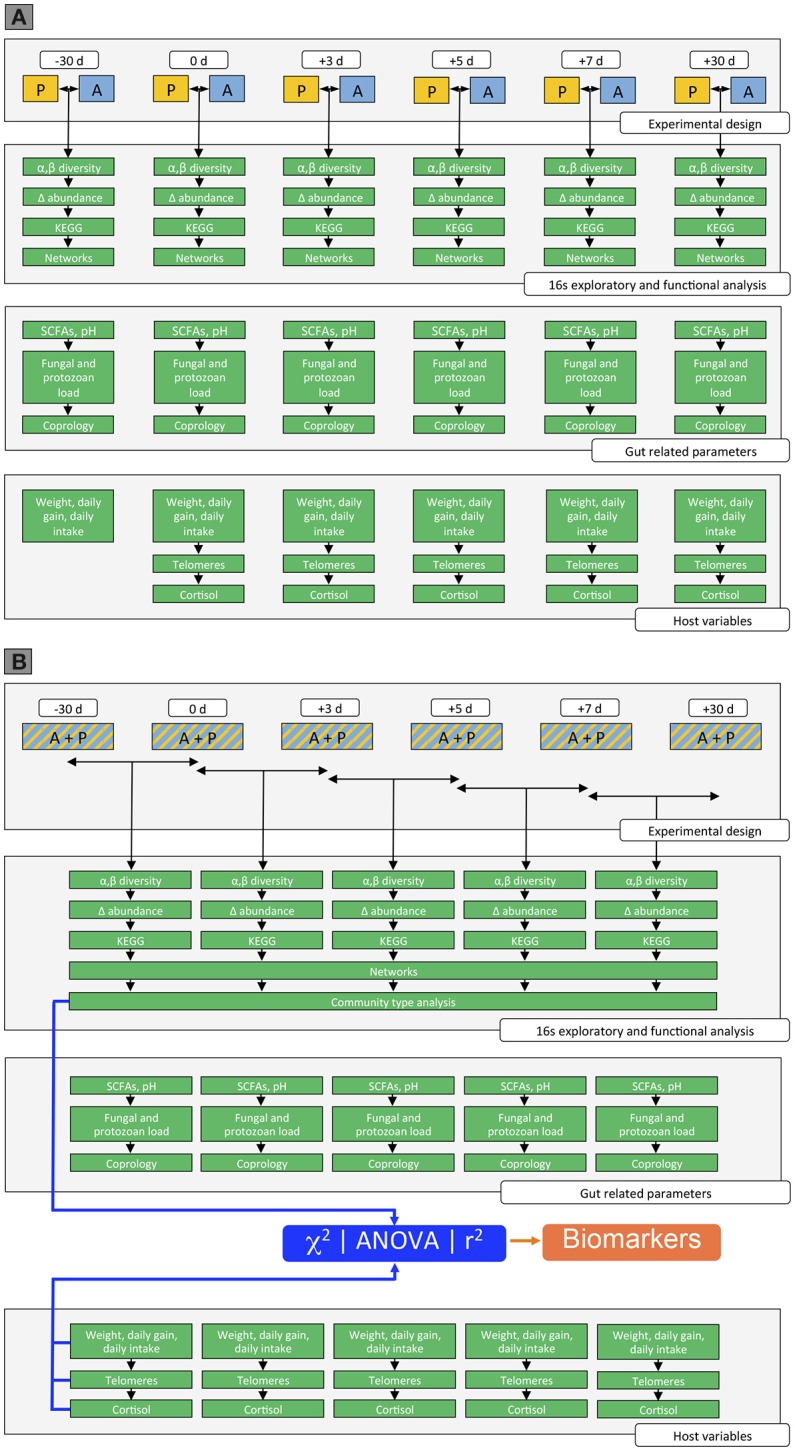
Overview of the data analysis in the study. **(A)** Effect of weaning method on gut microbiota composition, gut-related parameters, and host parameters across time. Step 1: Measurement of the gut microbiota composition between weaning methods across time. This step involved the analysis of the α-diversity and β-diversity between weaning methods across time, as well as the analysis to assess gut genera whose relative abundances changed between weaning methods across time, the determination of the corresponding KEGG pathways and the inference of the co-occurrence network. Step 2: Measurement of the gut related parameters between weaning methods across time. The gut parameters included the production of the SCFAs, the fungal, bacteria, and protozoan loads, as well as the number of parasite egg in the feces. Step 3: Measurement of the host parameters between weaning methods across time. The host parameters included the body weight, the daily average gain, the daily intake of concentrate and hay, as well as the telomere length and the salivary cortisol concentration. **(B)** Dynamics of the microbiota gut composition and function across time and their association with host phenotypic variables. Step 4: Measurement of the gut microbiota composition across time. This step involved the analysis of the α-diversity and β-diversity across time, as well as the analysis to assess gut genera whose relative abundances changed across time, the determination of the corresponding KEGG pathways and the inference of the co-occurrence network. Lastly, the analysis of the gut community types was performed across time. Step 5: Measurement of the gut related parameters across time. The gut parameters included the production of the SCFAs, the fungal, bacteria and protozoan loads, as well as the number of parasite egg in the feces. Step 6: Measurement of the host parameters across time. The host parameters included the body weight, the daily average gain, the daily intake of concentrate and hay, as well as the telomere length and the salivary cortisol. Step 7: Linking genera directly to gut related parameters and host phenotypes at different time points through the unsupervised gut community type, χ^2^, ANOVA, and Spearman correlation.

We used a multi-step approach to identify (i) the effects of the weaning methods on gut microbiota composition and host phenotypic variables across time; and (ii) the dynamics of microbiota gut composition and function after weaning and their association with the host phenotypic variables (Figure 2).

### The weaning method caused no changes in host performance but conferred significant effects on salivary cortisol

**Figure 3:**
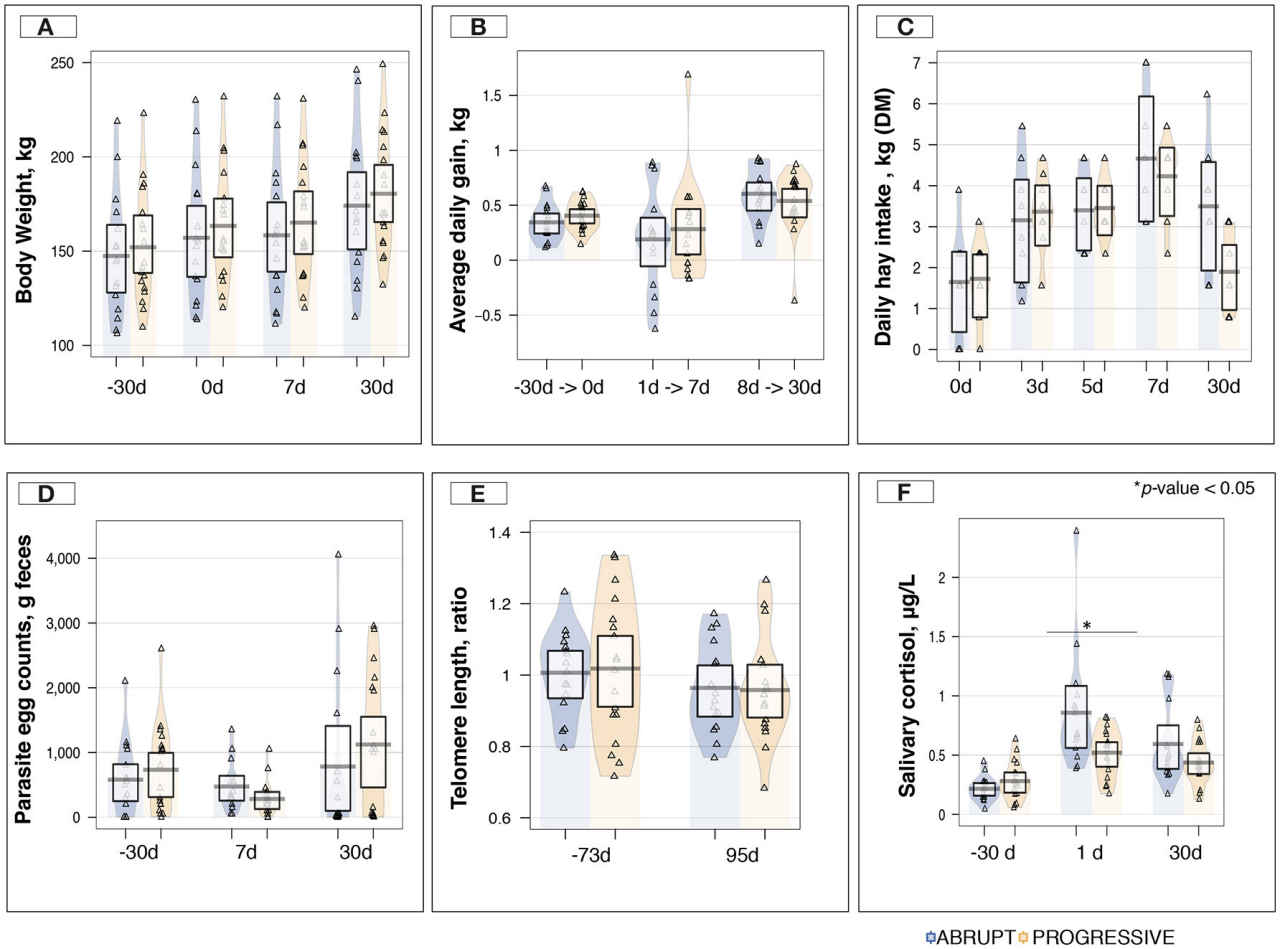
Host phenotypic variables between weaning methods and across time. **(A)** Boxplot and violin plot representation of body weight (kg) between weaning methods across time; **(B)** Boxplot and violin plot representation of average daily gain (kg) between weaning methods across time; **(C)** Boxplot and violin plot representation of daily hay intake per pen (kg of dry matter, DM) between weaning methods across time; **(D)** Boxplot and violin plot representation of parasite egg counts in feces (counts/g of feces) between weaning methods across time; **(E)** Boxplot and violin plot representation of the telomere length between weaning methods across time. The values reported in the plot correspond to the ΔΔCt between the Ct values of the amplified telomeric region and the Ct values of the single-copy gene (interferon-γ gene; IFGM) measured for each sample and relative to a control sample, in this case the value at day −73; **(F)** Boxplot and violin plot representation of saliva cortisol concentration (μg/L) between weaning methods across time. In all cases, progressive weaning is shown in orange color, abrupt weaning in blue, and values are overlaid as a triangle points; ^*^p < 0.05.

Body weight (Figure 3A) and average daily gain (Figure 3B) were not affected by the weaning method. Although total hay intake tended to be lower in the A group than in the P group during the first days post-weaning (Figure 3C), the average daily concentrate intake was similar across the two weaning methods, and no leftover feed at the concentrate feeders was recorded throughout the study (Table S4). Moreover, no significant changes in parasite fecal egg counts were observed across the two weaning methods (Figure 3D), and unchanged lengths of telomeres were observed before and after the experiment in both groups (Figure 3E). In contrast, a significant increase in salivary cortisol levels (*p* < 0.01) were observed in the A (0.85 ± 0.122 μg/L) compared to the P (0.52 ± 0.046 μg/L) group following the definitive weaning (Figure 3F). Further details on host response to the weaning method are shown in the Table S4.

### Microbiota composition was slightly affected by weaning method across time

The gut microbiota composition of foals that were progressively (P, *n* = 18) or abruptly (A, *n* = 16) weaned was analyzed over an extended period of 2 months through 16S rRNA gene sequencing (Figure 1).

A total of 10,844,916 paired-end 250 bp reads were obtained, 8,150,600 of which were retained as a high quality sequences (Table S5). These sequences were clustered into 10,868 OTUs. Among them, 8,103 were classified taxonomically down to the genus level (Table S5).

**Figure 4:**
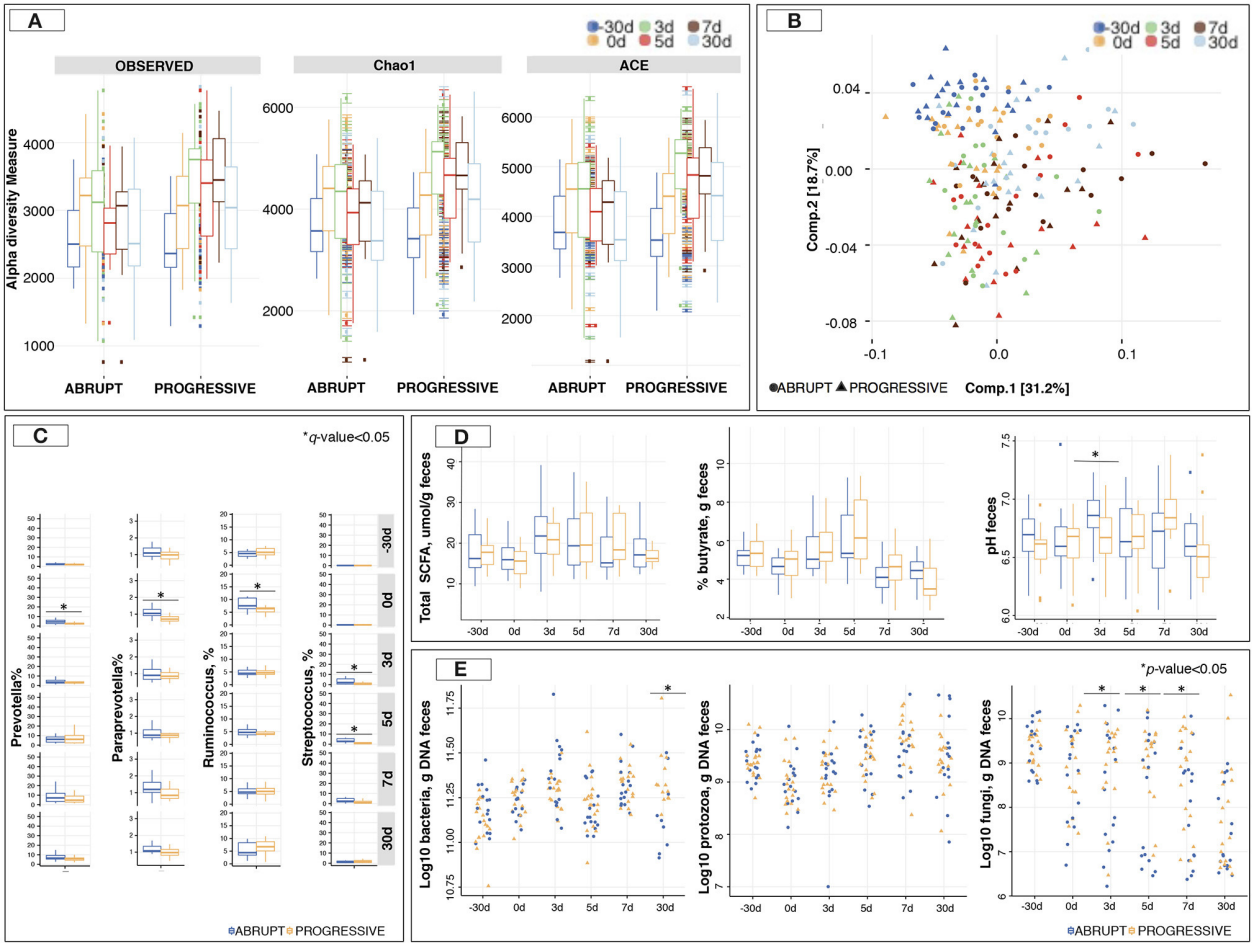
Gut microbiota composition and diversity between two different weaning methods across time. **(A)** Estimation of the richness and α-diversity indexes between weaning methods at different time points; **(B)** Principal Coordinate analysis of Unifrac distances to compare gut microbiota composition at the level of genera that differ between weaning methods across time. Both component 1 and 2 were plotted. Together they explained 49.9% of whole variation. Three outliers were removed from the graph; **(C)** Boxplot representation of genera significantly affected by weaning method across time; **(D)** Boxplot representation of total short chain fatty acids (SCFA; μmol/g feces), percentage of butyrate per g of feces, and pH in feces between weaning methods across time; **(E)** Boxplot representation of loads of bacteria, ciliate protozoa and anaerobic fungi in feces between weaning methods across time. In all cases, −30 days is shown in blue, 0 days in orange, 3 days in green, 5 days in red, 7 days in brown and 30 days post-weaning in light blue color. ^*^p or q < 0.05.

The microbiota richness and diversity based on Chao1 and Ace indexes were similar between weaning methods (Figure 4A). In terms of microbiota composition, the UniFrac distance followed by PCoA (Figure 4B) showed no distinct clustering between samples from the A and the P group, which was indicative of—if at all—no detectable differences in microbiota composition between the two weaning methods. This pattern was further confirmed by NMDS (Figure S2A) and sPLS-DA (Figures S2B,C). Similarly, the ANOSIM analysis suggested that the overall composition of the gut microbiota was largely similar between the animals of the two groups (*p* > 0.05) although it changed across time (*p* = 0.001; Figure S2D).

A more in-depth taxonomic analysis of bacterial genera using a Wilcoxon rank-sum test followed by multiple test correction revealed that the species belonging to *Prevotella, Paraprevotella*, and *Ruminococcus* were more abundant (*q* < 0.05) in the A group prior to weaning compared to the P group (Figure 4C; Table S6). The most conserved microbiota change after weaning was an increase of the species belonging to *Streptococcus* in the animals of the A group (*q* < 0.05) relative to the P group.

Despite the lack of systematic differences in gut microbiota composition in horses subjected to the two different weaning methods, we investigated putatively associated functional modifications. To this aim, we used PICRUSt, which predicts the gene content of a microbial community from the 16S rRNA gene survey by querying an existing database of microbial genomes. Most of the enzyme-level functional pathways were not significantly affected by any of the weaning methods. However, the A group displayed an enrichment (*q* < 0.05) of the glutamatergic synapse pathway, as well as amino acid metabolism (i.e., phenylalanine, tyrosine, tryptophan and lysine biosynthesis) before the weaning day (Table S7).

To understand how the gut microbiota functioned, SCFAs, pH measurements and the loads of fungi, protozoa and total bacteria in feces were investigated. SCFAs concentration showed no difference between the weaning methods at any time point (Figure 4D). Propionate, acetate and butyrate concentrations followed the same pattern (Table S4). However, the pH was lower (*p* < 0.05) in the P group than in the A group at 5 days post-weaning (Figure 4D). Interestingly, anaerobic fungal loads were lower (*p* < 0.05) in the A group relative to the P group, with more than 0.5 log of difference across all time points during the week following weaning (Figure 4E).

### Gut microbiota composition and functions shifts immediately after weaning

Irrespective of the effects of the weaning method on the gut microbiota, we next assessed the influence that definitive maternal separation at weaning (0 d) exerted on the composition of the gut microbiota of all the 34 foals. Thus, A and P animals were pooled as a group.

During the first week after weaning, gut microbiota underwent consecutive changes in composition and function until a relatively stable gut community was established at day 7 post-weaning in every foal. The most marked alterations were found at 3 days post-weaning, when the relative abundances of members of the genera *Prevotella, Oscillibacter, Streptococcus, Anaerovibrio, Lactobacillus* and of members of the family *Lachnospiracea incertae sedis* were significantly increased (*q* < 0.05; Figure S3A). At the same time, the relative abundances of the members of the genera *Fibrobacter, Clostridium* XIVa*, Ruminococcus, Treponema* and of the members of the as yet unclassified family *Lachnospiraceae* were significantly decreased (*q* < 0.05, Figure S3A). The complete list of increased and decreased genera including direction, coefficient and *q*-values is presented in Table S8.

PICRUSt was used to gain a better understanding of the functional implications of these compositional changes following maternal separation at weaning. The RIG-I like receptor signaling, *Staphylococcus aureus* infection, apoptosis, as well as ion channel functions and ether lipid metabolism pathways were significantly overrepresented after maternal separation (Figure S3B, Table S9). Conversely, steroid biosynthesis, α-linolenic acid metabolism, ubiquitin system and isoflavonoid biosynthesis were significantly underrepresented following maternal separation at weaning (Figure S3B, Table S9).

The concentration of SCFAs in feces significantly increased following the definitive maternal separation at weaning (Figure S3C), but not the fecal pH (Figure S3D).

### The gut microbiota composition during the first 3 days post-weaning is highly variable between individuals: community types

**Figure 5:**
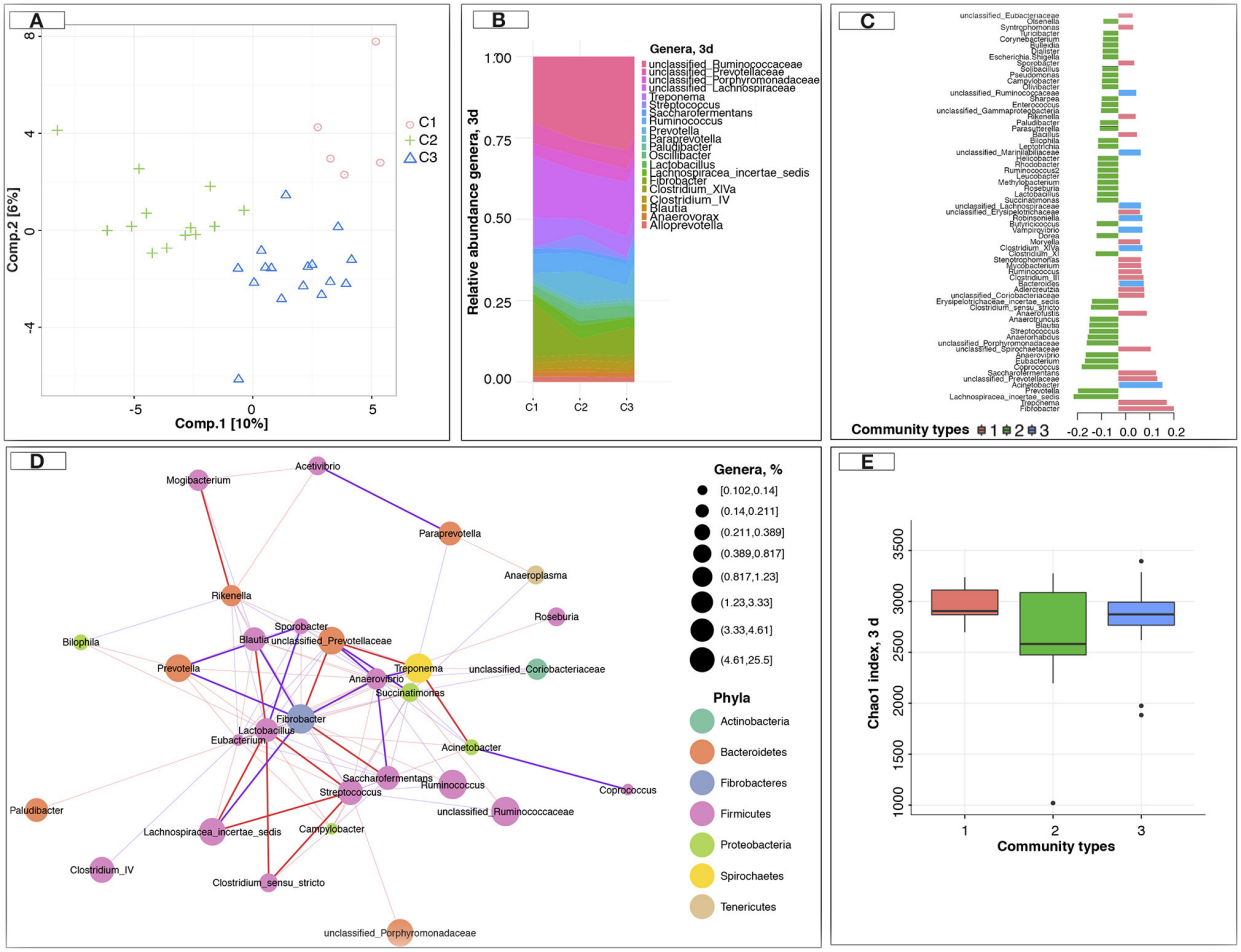
Dynamics of the gut microbiota composition at 3 days post-weaning. **(A)** sPLS-DA of gut microbiota composition at the level of genus at 3 days post-weaning; **(B)** Genera that dominant in the different gut community types at 3 days post-weaning; **(C)** sPLS-DA contribution plot at 3 days post-weaning, which displays the importance of each genera in the sPLS-DA model and in which gut community type they were the most abundant (contrib = “max”), according to the median (method = “median”); **(D)** Co-occurrence network at 3 days post-weaning. The correlations among genera were calculated using the REBECCA method (Ban et al., 2015). The size of the node is proportional to genera abundance. Node fill color corresponds to phylum taxonomic classification. Edges color represent positive (red) and negative (blue) connections, the edge thickness is equivalent to the correlation values; **(E)** Estimation of the Chao1 index at 3 days post-weaning between community types (or clusters). In all cases, pink color represents the community type 1, green color represents the community type 2, and blue color represents the community type 3.

Having established that the gut microbiota composition and function appeared to be markedly disrupted during the first days post-weaning, we subsequently investigated the intra- and inter-individual variation across time for all 34 foals. The intra- and inter-individual variation was studied based on the conceptual framework of enterotypes, or more generically, community types, according to which the samples are clustered into bins based on their taxonomic similarity. Instead of observing a stable number of gut community types across the six time points under study, we observed that the number of gut community types dynamically changed with respect to weaning (Figure S4). The most substantial changes in gut community types occurred at day 3 post-weaning (Figure S4). At this time point, we found evidence for three gut community types (Figure 5A). Community type 1 displayed the highest levels of *Acinetobacter, Adlercreutzia, Bacillus, Fibrobacter, Rikenella*, and *Treponema*, but lower levels of *Eubacterium, Anaerovibrio, Blautia, Clostridium* XI, *Coprococcus, Lachnospiracea incertae sedis*, and *Prevotella* (Table S10). Community type 2, on the other hand, showed the opposite pattern. Eventually, community type 3 appeared as a gradient of dominant taxa between community type 1 and type 2 (Figure 5B). The shift from community type 1 to community type 2 coincided with the transition from milk to cereal-based diet 1 month prior weaning. In fact, community type 1 was found in 80% of suckling foals (−30 d) and almost disappeared following weaning, while community type 2 only appeared after weaning (Figure S4).

It was not only the most abundant genera that differentiated the gut community types (Figure 5C); rather, the community types were identified based on complex configurations of numerous genera that might engage in cooperative metabolism (positive interaction) or competition (captured by the number and magnitude of mutually negative interactions). That is, the co-occurring *Acinetobacter, Fibrobacter*, and *Treponema* were negatively associated with members of the Firmicutes phylum (i.e., *Blautia, Coprococcus, Eubacterium*, and *Lachnospiraceae incertae sedis*, Figure 5D). Although the gut community types did not differ in their respective bacterial richness (Figure 5E), the predicted functional capacity of each community type was distinct. Community type 2 presented an enrichment of the carbohydrate digestion and absorption pathways, as well as of the galactose metabolism, ether lipid metabolism, and secondary bile acid biosynthesis compared to the other two community types at 3 days post-weaning (Table S11). Across all the examined pathways related to the immune system response, the RIG-I-like receptor-signaling pathway was enriched in community type 2 when compared to the other ones.

Given the differences of predicted functional capacity between considered gut community types at 3 days post-weaning, it is not surprising that five out of the eight SCFAs measured, namely acetate, propionate, butyrate, isovalerate, and valerate were significantly higher (*p* < 0.05) in the animals belonging to community type 2 than in the other two communities at 3 days post-weaning (Figures S5A,B, Table S12). We also observed that animals with lower loads of fungi but higher loads of bacteria and protozoa at 3 days post-weaning were likely to belong to community type 2 (*p* < 0.05, Figures S5C,E, Table S12).

Because gut microbiota composition may also play a key role in controlling the physiological and neurophysiological mechanisms involved in the weaning stress response, we investigated putative associations between stress indicators (telomere length and salivary cortisol levels) and the different gut community types. Of note, cortisol levels were lower in community type 2 and 1 relative to community type 3 after weaning (Figure S5F). Salivary cortisol concentrations in response to weaning were 0.40 ± 0.19, 0.48 ± 0.7, and 0.59 ± 0.25 μg/L in community type 1, 2, and 3, respectively (Figure S5F). On the contrary, the telomere length was higher (*p* < 0.05) in community type 2 and 3 compared to community 1 at 3 days post-weaning (Figure S5G).

Furthermore, average daily gain and the number of times going to the concentrate feeder was greater in community type 2 relative to community type 3, especially during the first week after weaning (Figures S5H,I). We next studied the association between the fecal egg counts, which are an indirect measure of the burden of adult cyathostomin stages, and the community type at 3 days post-weaning. Community 1 type was associated with the lowest fecal egg counts on average at 7 days (Figure S5J).

Then, we assessed the contribution of different putative drivers on gut community types at 3 days post-weaning, like for example differences in weaning method, gender, pen or genetic background. No significant difference (*p* > 0.05) in gut community types was detected between weaning method, foals' gender, foals' age, or cohousing animals, i.e., foals cohabitating in the same pen (Figure S4). Intriguingly, 80% of the individuals from community type 1 at 3 days post-weaning presented higher genetic relatedness (as they were siblings, Figure S1), so that the abundance of bacteria belonging to community 1 were more similar between genetically similar individuals than genetically distant ones.

### Linking genera directly to host phenotypes rather than relying on unsupervised gut community types at 3 days post-weaning gives similar results

**Figure 6:**
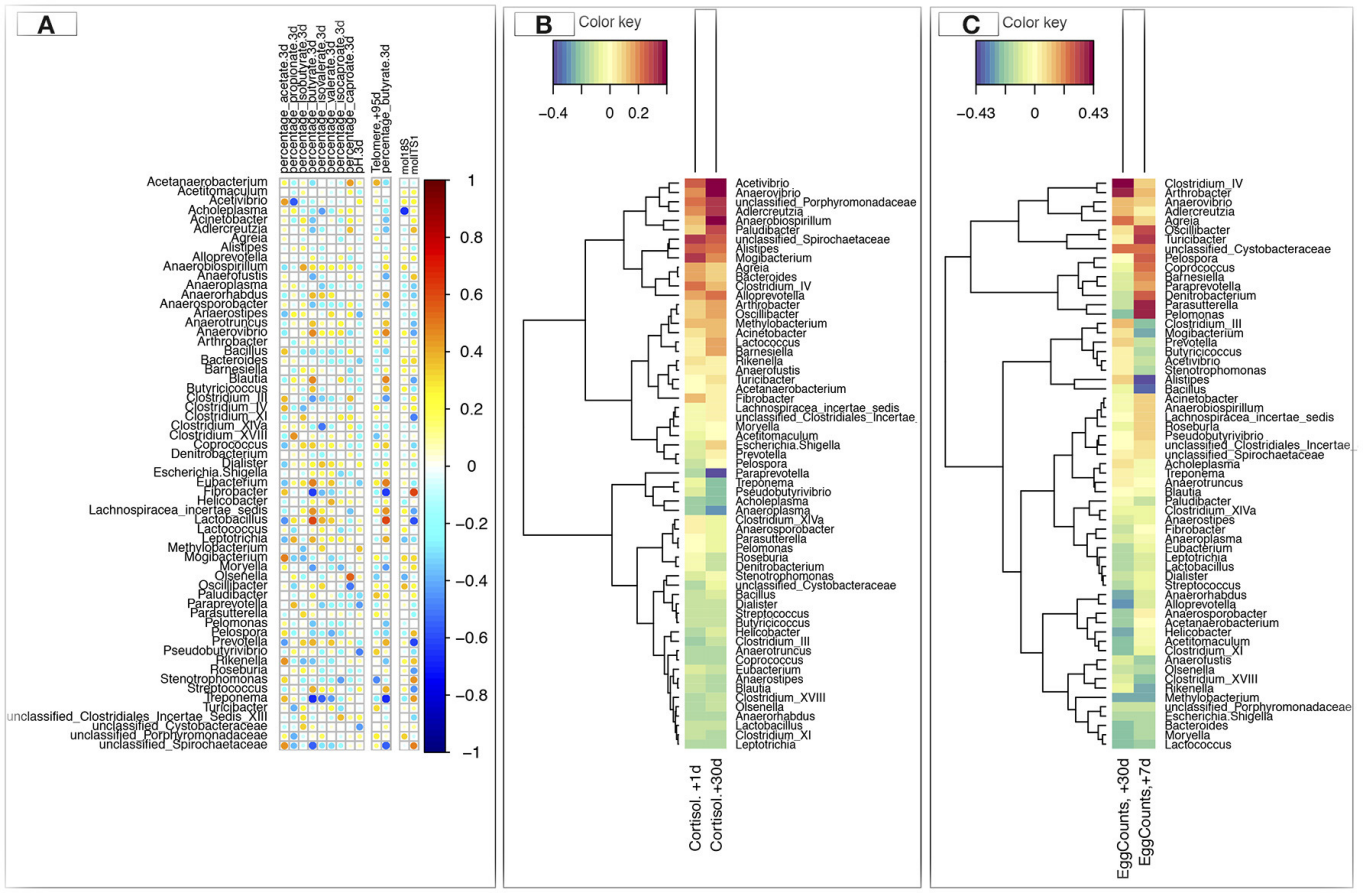
Determination of potential key genera for performance and stress parameters in foals at 3 days post-weaning. **(A)** Spearman's correlation heatmap that represents the significant correlation values between the relative abundance of gut bacterial genera and percentage of different short chain fatty acids (SCFA) in feces, pH, telomere length, and loads of protozoa (log 10 molecules/g DNA feces) and fungi (log 10 molecules/g DNA feces) in feces at 3 days post-weaning. Values reported for telomere lengths correspond to ΔΔCt between Ct value of telomere amplification and the single-copy interferon-γ (IFGM) amplification for each sample relative to a control sample, here the value at day −73; **(B)** Spearman's correlation heatmap that represents the correlation values between the relative abundances of gut genera at 3 days post-weaning and saliva cortisol levels (μg/L); **(C)** Spearman's correlation heatmap that represents the correlation values between the relative abundances of the gut genera at 3 days post-weaning and parasite egg counts (eggs/g feces). In all heatmaps, red color represents positive association and blue color represents negative association.

Since discrete gut community types might be less effective to discover associations between an individual's gut function, performance and his gut microbiota composition, we used Spearman correlation to translate gut microbiota composition to functionality at the host level at 3 days post-weaning. These analyses revealed that the individuals showing the highest levels of *Blautia, Clostridium* XI*, Coprococcus, Eubacterium*, and *Lactobacillus* spp. at 3 days post-weaning were more likely to present a higher percentage of N-butyrate in feces (Figure 6A). They exhibited longer telomere length (Figure 6A), but lower cortisol levels in saliva (Figure 6B), lower fecal egg counts (Figure 6C) and lower loads of commensal fungi. Conversely, individuals with higher levels of *Acinetobacter, Adlercreutzia, Bacteroides, Fibrobacter*, and *Rikenella* spp. at 3 days post-weaning showed positive association with the concentrations of cortisol in saliva and fungal loads and negative association with telomere length and butyrate percentage in feces (Figures 6A,B). Genera such as *Acetivibrio, Anaerovibrio*, and *Alistipes* were also positively associated with salivary cortisol release. Furthermore, a significant positive correlation was found between bacteria such as *Clostridium* IV*, Coprococcus, Anaerovibrio, Agreia, Oscillibacter, Turicibacter*, and unclassified *Cystobacteraceae* and parasite egg counts. *Bacteroides* was negatively correlated with parasite egg burden (Figure 6C).

## Discussion

In this study, host phenotypic variables and gut microbiota composition were analyzed in relation to two different weaning methods in foals. Based on salivary cortisol levels, the degree of stress was clearly higher in A than in P immediately after the definitive maternal separation. However, salivary cortisol concentrations were lower than those reported by Erber et al. (2012) in weaned foals, probably because ponies are less excitable than sport horses. Remarkably, the cortisol production and release did not exert detrimental effects on animal growth, parasite burden and the overall dry matter intake.

Additionally, our study shows that the weaning method affected just a few gut microbial species in foals supplemented with the same diet and kept under the same environmental conditions, namely *Paraprevotella, Prevotella, Ruminococcus*, and *Streptococcus*, as well as fungi loads.

One month before weaning (coinciding with the introduction of cereal-based diet), A group displayed higher abundance of *Prevotella, Paraprevotella*, and *Ruminococcus*, which possess saccharolytic and fibrolytic capacities, respectively (Daly et al., 2001). The presence of higher cereal-adaptable species before weaning in A group suggested that their gut microbiota was primed for weaning compared to P, perhaps because separating mares and foals transiently before weaning led to sustained release of stress hormones in P group and thus, inhibit the proliferation of the aforementioned genera. In fact, a pilot study carried out by Erber et al. (2012) provides support to this hypothesis. These authors reported that cortisol release was elevated and sustained over a longer time in foals weaned progressively relative to those weaned abruptly.

*Streptococcus*, on the other hand, showed an opposite pattern after weaning, its abundance being lower in the P animals. *Streptococcus* species are widely regarded as being commensal or commensal-like organisms in horses (Costa et al., 2015; Ericsson et al., 2016), although *Streptococcus equi* and *S. zooepidemicus*, opportunistically induce diseases in situations of stress in horses (Timoney, 2004; Pelkonen et al., 2013). Additionally, evidence shows that *Streptococcus* spp. are able to generate the neurotransmitter serotonin (5-hydroxytryptamine, 5-HT, Cryan and Dinan, 2012), which plays a central role in the gut motor function and digestion, as well as in various cognitive and mood disorders, including sleep, drowsiness and central fatigue (Best et al., 2010). Reduced levels of 5-HT or its precursor L-tryptophan have been described in patients suffering from major depressive disorder (Fakhoury, 2016) and 5-HT depletion in *Drosophila* induced similar symptoms as observed in depression (Ries et al., 2017). Because it was impossible to assign sequences to the level of species, the potential effects of the *Streptococcus* proliferation on the host after weaning, and whether species were pathogenic or capable to synthesize 5-HT levels remain unclear and need further evaluation.

Immediately after weaning, anaerobic commensal fungal loads were also higher in P group than in A group, likely due to their higher intake of hay. In ruminants, higher commensal anaerobic fungal loads were ascribed to greater amount of complex, cellulose-rich material to degrade in the rumen (Bauchop, 1981; Wei et al., 2016). Therefore, analogous mechanisms are thought to be found in horses, which are hindgut fermenters.

Our study extends the findings of others authors who studied the alterations in the microbiota composition in the gut of foals after maternal separation at weaning (Faubladier et al., 2014; Costa et al., 2016). Moreover, it demonstrates, for the first time, the contribution of the gut microbiota composition following maternal separation at weaning to horse phenotypes.

Maternal separation at weaning immediately shifted the composition of the gut microbiota in all animals, revealing fitness differences among species. It is likely that these differences were linked to the maternal separation-associated stress (Waran et al., 2008). The induced stress could act either directly by killing certain beneficial species (Mach and Clark, 2017), inducing the proliferation of pathobionts and the expression of virulence genes (Fang et al., 2016) or, indirectly, by modifying the gut environment that supports them (Eisenstein, 2016). Along with our results, Bailey et al. (2011), using a model of social disruption among adult mice, contended that exposure to stress resulted in a substantial decrease of *Bacteroides* spp. compared to their levels in control mice, but an increase in the relative abundance of *Clostridium* spp. Similarly, O'mahony et al. (2009) reported that feces of adult mice that had undergone maternal separation for 3 h per day from postnatal days 2–12 presented an altered microbiota composition when compared with the non-separated control animals. Less probable, is the impact of milk withdrawal on gut microbiota composition at weaning. Indeed, milk consumption at that age is low (<1 L per day), as milk yield in mares dramatically drops after the first 3 months of lactation (Doreau et al., 1990). Moreover, at this age, suckling was combined with cereal-based diet and forage intake, because foals eat forage from the second day of life (Faubladier et al., 2013). As a consequence, we expect milk dry out at weaning to have only had a superficial impact on gut microbiota shifts as observed elsewhere (Faubladier et al., 2014).

Having established that the gut microbiota composition and function appeared to be markedly shifted after weaning in all animals, we subsequently investigated its inter-individual variation following the suggestion by Arumugam et al. (2011). Our time series data, which extended for a period of 2 months, demonstrated that the gut microbiota could be partitioned into different community types across time. However, gut community types were not stable over the time. Notably, the most substantial shift in gut microbiota community types occurred at day 3 post-weaning. As observed in other species (Kittelmann et al., 2014; Mach et al., 2015), the different gut community types displayed smooth abundance gradients of key genera. While trying to understand the factors driving the gut microbiota community types across time, we found evidence that 80% of the animals belonging to the community type 1 at day 3 post-weaning were genetically correlated. Because individuals were not sharing their pens, similarities between the microbiota of related individuals could reflect host genetic relatedness. However, this finding was not reproducible for community types 2 and 3, suggesting that factors other than host genetics (i.e., environmental and stochastic factors) are shaping the gut microbiota community type following weaning.

Interestingly, microbiota gut composition appears to be specifically associated with different host phenotypes in humans (Arumugam et al., 2011; Wu et al., 2011; Koren et al., 2013; Vandeputte et al., 2016), chimpanzees (Moeller et al., 2012), mice (Hildebrand et al., 2013), pigs (Mach et al., 2015; Ramayo-Caldas et al., 2016), and ruminants (Kittelmann et al., 2014).

Significant associations between several genera and host salivary cortisol levels were found following weaning. For example, abundance of *Eubacterium, Coprococcus, Clostridium* XI, *Blautia*, and *Lactobacillus* (all of them primarily found in community type 2) were negatively associated with host salivary cortisol, whereas other genera (i.e., *Escherichia, Acetivibrio, Anaerovibrio*, and *Alistipes*) were positively associated with salivary cortisol release. The same pattern characterized by increased cortisol secretion, increased abundance of Proteobacteria such as *Escherichia* and reduced number of *Lactobacillus* was found in infants with exposed prenatal stress (Zijlmans et al., 2015) and in rhresus monkeys prenatally exposed to a very different type of stressor (acoustic stress; Bailey et al., 2004). Moreover, a study in humans also showed a progessive increase in *Bacteroidaceae* and salivary cortisol levels during a stressful event (1-day examination; Kato-Kataoka et al., 2016). Although as yet hypothetical, the mechanisms by which cortisol release might alter gut microbiota compositions range from stress-induced changes in intestinal physiology that modify microbial niches (i.e., alteration of gut permeability and barrier function or bile acid concentrations) to the influence of interbacterial signaling, growth and virulence (Cryan and Dinan, 2012).

**Figure 7:**
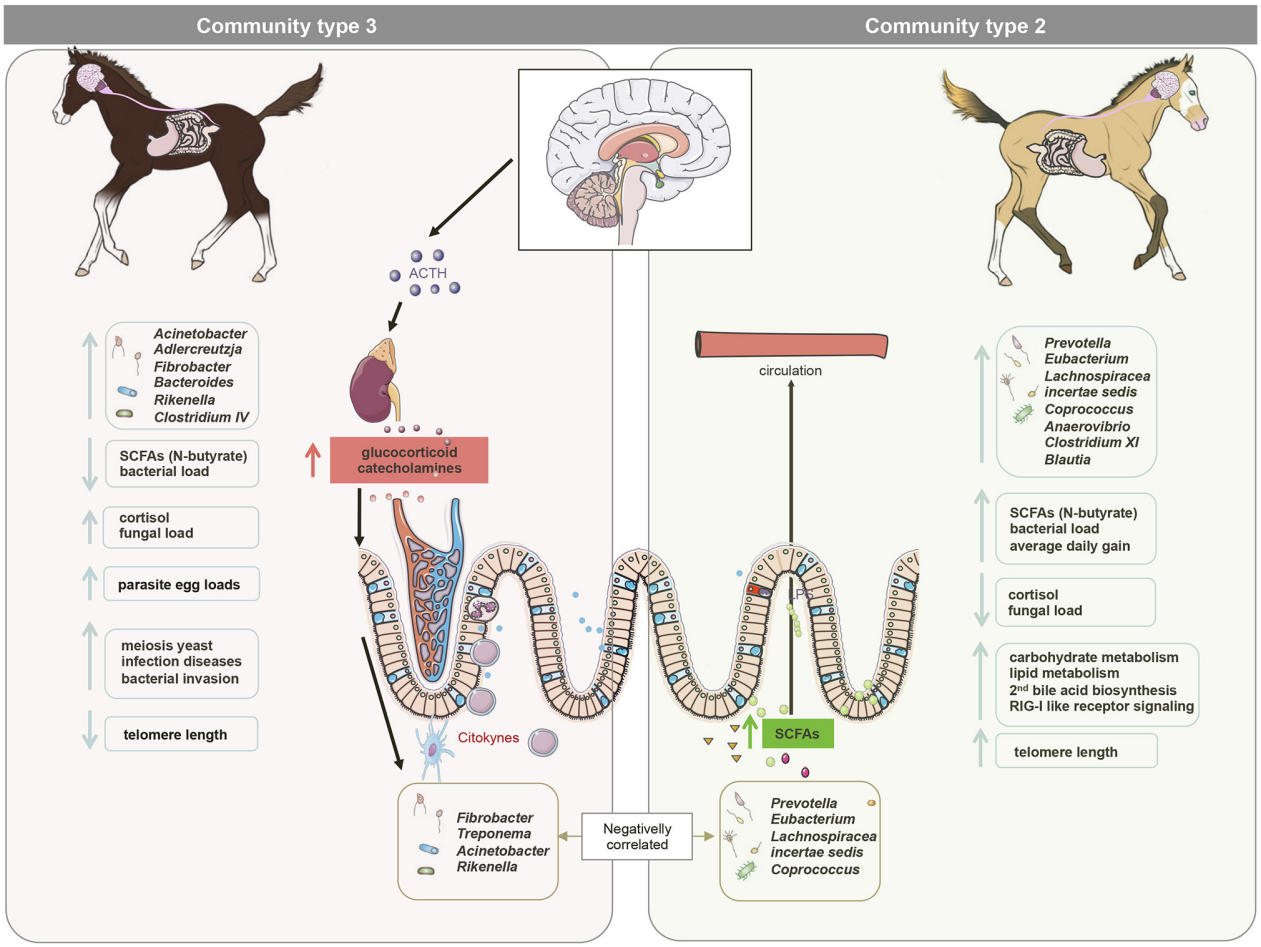
A model for gut microbiota modifications and their effects on host physiology after maternal separation at weaning. We hypothesize that maternal separation at weaning increases the release of cortisol in the peripheral viscera, including the gut. Higher cortisol release induced alterations in bacterial-fungal inter-kingdom, increasing the abundance of *Acinetobacter, Adlercreutzia, Fibrobacter, Bacteroides, Rikenella*, and *Clostridium IV* (mainly dominant in the gut community type 3) and the overall loads of fungi and parasite egg counts in the feces. It is therefore likely that these alterations acted as a priming stimulus for the gut ROS and RONS production in the gut, leading to DNA damage and telomere length shortening. Conversely, bacteria such as *Eubacterium, Coprococcus, Clostridium XI*, and *Blautia* (dominant in the community type 2) were found to be negatively correlated with cortisol levels in saliva, but positively correlated to telomere length and the N-butyrate at 3 days post-weaning, suggesting a possible effect of N-butyrate on the protection of telomere length. The higher N-butyrate production was accompanied by higher average daily gain in almost all animals, suggesting a positive impact of these metabolites on the productivity of the host. This figure was produced using Servier Medical Art, available from http://www.servier.com/Powerpoint-image-bank.

Among the aforementioned bacteria primarily belonging to community type 2, there were also strong and positive associations between their abundance, N-butyrate levels and telomere length at 3 days post-weaning. In support of this association, Sheridan et al. (2016) showed that the predominant butyrate-producing bacteria belong to the phylum Firmicutes and include *Eubacterium* and *Roseburia* spp. Butyrate might influence several physiological processes such as cell signaling, neurotransmitter synthesis and release, mitochondrial function and oxidative stress (reviewed by Clark and Mach, 2017). Although other factors undoubtedly contribute to telomere length, it might be suggested that butyrate-producing bacteria protect from telomere damage (Figure 7). This later protection may be attributable to the influence of N-butyrate on the activity of the cyclooxygenase 2 (COX-2), which detoxifies and reduces H_2_O_2_ and reactive oxygen species levels (Blachier et al., 2007; Mottawea et al., 2016).

The higher N-butyrate production at 3 days post-weaning was coupled to an increase in propionate production in almost all animals, which partakes in many crucial aspects of host energy production and metabolism (Nicholson et al., 2012). Given that ~65% of the net energy requirement of horses is supplied by SCFAs (Al Jassim and Andrews, 2009), these differences in SCFA production possibly explained the greater average daily weight gain in the individuals belonging to community type 2. Concomitantly to these clear differences at the SCFAs, we observed an enrichment of carbohydrate metabolism, secondary bile acid biosynthesis and RIG-I-like receptor signaling pathway, which functions in the pattern recognition of bacterial and viral pathogens (Medzhitov, 2007), in the community type 2 compared to the other two communities at 3 days post-weaning. Therefore, it is possible that community type 2 confers better response adaptation to weaning (Figure 7).

Using the egg-burden data, we highlighted that Clostridiales such as *Clostridium* IV and *Coprococcus* were significantly associated with parasite egg counts, whereas *Bacteroides* was negatively associated with parasite egg counts. Taken together, these data suggest that the initial parasite infection was able to determine changes in the abundance of some specific genera as already outlined in other host-parasite models (Walk et al., 2010; Holmes et al., 2011; Li et al., 2012; Wu et al., 2012; Lee et al., 2014; Osborne et al., 2014; Holm et al., 2015). Our findings are particularly well corroborated by a recent study demonstrating that helminth infection promotes the expansion of Clostridiales communities (i.e., *Clostridium* clusters IV, XIVa, and XVIII and Erysipelotrichales strains) that outcompete Bacteroidales communities and reporting that *Coprococcus* and *Bacteroides* in particular, were positively and negatively associated with changes in egg burden (Ramanan et al., 2016). This led to the hypothesis that deworming treatments reduce the protective levels of Clostridiales and increase the pro-inflammatory Bacteroidales, which support the model of the hygiene hypothesis (reviewed by Clark and Mach, 2016).

Altogether, the correlative nature of associations between outcomes from which causality cannot be determined limits the interpretation of our results. Therefore, it is of paramount importance to carry on larger longitudinal studies to explore the causes and the persistency of these interactions.

Despite these limitations, our study showed that the overall host variables and the composition of gut microbiota were largely similar between progressively and abruptly weaned foals, although few specific species were modified across time. For instance, genera belonging to *Prevotellaceae* family and *Ruminococcus*, frequently referred to as beneficial bacteria, were less abundant during the progressive weaning, suggesting that the gut microbiota in the P cohort was less adapted to weaning. *Streptococcus*, on the other hand, showed the opposite pattern after weaning; although there is little information know about *Streptococcus* role in horses at weaning. However, one of the potential benefits afforded by progressive weaning was the increase of anaerobic fungal loads, which are thought to increase the capacity for fermenting the complex polysaccharides from diet.

Regardless of the particular effects of weaning method, maternal separation at weaning shaped gut microbiota during the first days post-weaning in a manner that correlates with alterations in host phenotypes (i.e., salivary cortisol, telomere lengths, parasite egg counts, and performance). Although causality could not be determined from correlations, we highlighted potential microbial biomarkers that could predict the likelihood for adaptation after weaning and provided novel insights into the regulatory mechanisms that control physiological adaptations to weaning in horses. Further experiments are needed to understand whether the alterations of gut microbiota at weaning might affect the neurobiology of stress and endocrine function of the microbiota, as suggested by Cryan and Dinan (2012), as well as the host in the long-term.

## Author contributions

NM designed and carried out the bioinformatics and biostatistical analysis, wrote the main manuscript text, and prepared all the figures. AF performed the cortisol and telomere length analysis. FR organized the samplings, fed the animals and handled the whole experiment in the experimental station at Nouzilly. MB and MM carried out the design of the RT-qPCR analyses. MM created the Figure 2. JR extracted the DNA and performed most of the RT-qPCR. DE prepared the libraries and performed the MiSeq sequencing. GS performed analyzed the parasite eggs counts in feces. SK, MM, MB, GS, PG, MPM, and LL helped to interpret data. LL and MPM designed the study. All authors reviewed the manuscript and approved the final version.

### Conflict of interest statement

The authors declare that the research was conducted in the absence of any commercial or financial relationships that could be construed as a potential conflict of interest.

## Supplementary material

Table S1 Ingredient and nutrient composition of the foals' experimental concentrate.

Table S2 Summary of the study samples and fecal bacterial 16S rRNA gene amplicon sequence parameters.

Table S3 Sequences of the gene primers used for the qPCR of bacterial, fungal and protozoan loads.

Table S4 Differences in pH, short chain fatty acids (SCFA), body weight, concentrate, and hay intake, as well as parasite egg counts, salivary cortisol, and telomere lengths between weaning methods across time.

Table S5 The OTU abundance and annotation between weaning methods across time. The OTU abundance is listed by animal and time, including sequence number, abundance, and closest reference strain and similarity.

Table S6 Differences in relative abundance of genera between weaning methods across time. We performed a Kruskal–Wallis test with the Benjamin–Hochberg multiple correction hoc test. The average percentage of each genus in each time point and weaning method is indicated.

Table S7 Differences in KEGG pathway abundance between weaning methods across time.

Table S8 Differences in the relative abundance of gut genera across time. We performed a Kruskal–Wallis test with the Benjamin–Hochberg multiple correction hoc test. The average percentage of each genus in each time point is indicated. Genera with relative low abundance that were invariable between time points are not detailed.

Table S9 Differences in KEGG pathway abundances across time.

Table S10 Relative abundance of genera between gut community types at 3 days post-weaning.

Table S11 Differences in KEGG pathway abundances between gut community types at 3 days post-weaning.

Table S12 Link between the three gut community types defined at day 3 post-weaning, the gut fermentation parameters and the host parameters.

Figure S1 Genetic resemblances between the 34 foals in the experiment **(A)**. Pedigree plot. A six-generation pedigree plot is illustrated, with different shapes for male (squares) and female (circles). The shapes are black for the 34 foals in the study. **(B)** Heatmap of the kinship coefficient matrix, which assess the genetic resemblance between individuals. Each entry in the matrix is the kinship coefficient between two subjects. Animals are arranged in the order of their genetic relatedness; genetically similar animals are near each other. Note that the diagonal elements did not have values above unity, showing no consanguineous mating in the families. Animals pertaining to progressive weaning are colored in orange. The community type at 3 days post-weaning is delineated next to the animal name. At 3 days post-weaning, the distribution of gut community types displayed between individuals with higher genetic relatedness was equal (1:1:1 of Community1: Community 2:Community3). However, 80% of the individuals from the community type 1 were siblings.

Figure S2 Multidimensional reduction methods for elucidating the effects of weaning method and time on gut microbiota composition. **(A)** Correspondence analysis of Unifrac distances to compare gut microbiota composition at the level of genus across time. Both axes 1 and 2 were plotted. Together they explained 50.9% of whole variation. The point's shape indicates the type of weaning (round: brutal; triangle: progressive); **(B)** sPLS-DA of gut microbiota composition at the level of genus across time; **(C)** Contribution plot of sPLS-DA model of each genera across time. The plot displays the importance of each genus in the sPLS-DA model and in which time point they are the most abundant (contrib = “max”), according to the median (method = “median”); **(D)** ANOSIM results to test for differences in gut microbiota composition at the level of genus between weaning methods across time. The analysis showed an *R* = 0.253 (*P* < 0.001) across time, indicating that all samples within a time point were more similar to each other than to any other samples from different time points. In all cases, −30 days is shown in blue, 0 days in orange, 3 days in green, 5 days in red, 7 days in brown and 30 days post-weaning in light blue color.

Figure S3 Genera and KEGG pathways abundance across time. **(A)** Boxplot representation of the most abundant genera that significantly shifted after maternal separation at weaning; **(B)** The KEGG pathways differentially expressed across time (*q* < 0.01) and identified by PICRUSt tool were displayed using a heatmap. The subpathways were also represented in the lateral bar using different colors; **(C)** Boxplot representation of total short chain fatty acids concentration (μmol/g feces) across time; **(D)** Boxplot representation of fecal pH across time. In all cases, −30 days is shown in blue, 0 days in orange, 3 days in green, 5 days in red, 7 days in brown and 30 days post-weaning in light blue color.

Figure S4 Dynamisms of the gut microbiota community types across time. In the cell plot, each column indicates one different time points, while each row displays the gut community type attributed to each animal. In all cases, pink color (community type 1), green color (community type 2), and blue color (community type 3). For each row in the cell plot, the information concerning the animal ID, weaning method, sex, sire, mare, and body weight at birth are depicted.

Figure S5 Effect of community type on performance and health parameters at 3 days post-weaning. **(A)** Boxplot representation of propionate (μmol/g feces) concentration between the three gut community types; **(B)** Boxplot representation of butyrate concentration (μmol/g feces) distribution between the three gut community types; **(C)** Boxplot representation of fungal loads (log 10 molecules, g of DNA in feces) between the three gut community types; **(D)** Boxplot representation of bacterial loads (log 10 molecules, g of DNA in feces) between the three gut community types; **(E)** Boxplot representation of protozoan loads (log 10 molecule, g of DNA in feces) between the three gut community types; **(F)** Boxplot representation of saliva cortisol concentration (μg/L) between the three gut community types; **(G)** Boxplot representation of the telomere length between the three gut community types relative to a control sample. The values reported in the plot correspond to the ΔΔCt between the Ct values of the amplified telomeric region and the Ct values of the single-copy gene (interferon-γ gene; IFGM) measured for each sample and relative to a control sample, in this case the value at day −73; **(H)** Boxplot representation of average daily gain (Kg/d) between the three gut community types; **(I)** Boxplot representation of frequency eating concentrate per day between the three gut community types; **(J)** Boxplot representation of parasite eggs counts (eggs/g feces) distribution across the three gut community types. In all cases, pink color represents the community type 1, green color represents the community type 2, and blue color represents the community type 3. ^*^*p* < 0.05.

## Abbreviations

ANOSIM: Analysis of similarities
ANOVA: Analysis of the variance
COX-2: Cyclooxygenase 2
CSS: Cumulative sum scaling
DM: dry matter
HPA: hypothalamic pituitary adrenal axe
INRA: National Institute for Agricultural Research
IFGM: interferon-γ
KEGG: Kyoto Encyclopedia of Genes and Genomes
NMDS: Non-Parametric Multidimensional Scaling
OUT: Operational Taxonomic Unit
PCoA: Principal component analysis
QIIME: Quantitative insights into microbial ecology
qPCR: real time quantitative PCR
RDP: Ribosomal Database Project
SAM: sympato-adrenomedullary axe
SCFA: short chain fatty acids
sPLS-DA: sparse partial least squares discriminant analysis
5-HT: 5-hydroxytryptamine.
