# Supplementary material for: The Effects of Weaning Methods on Gut Microbiota Composition and Horse Physiology
Source: Front Physiol. 2017 Jul 25;8:535. doi: 10.3389/fphys.2017.00535 (PMC5524898; doi:10.3389/fphys.2017.00535)
Supplement: Table S1 — Ingredient and nutrient composition of the foals' experimental concentrate. [file Table1.DOCX]

**Table S1**. Ingredient and nutrient composition of the foals’ experimental concentrate.

| Item |  |
| --- | --- |
| Ingredients | % of MS |
| Oat grain | 90.82 |
| Soybean hulls | 9.18 |
| Nutrients |  |
| ME, Mcal/kg | 3.44 |
| CP, % of DM | 13.26 |
| Ether extract, % of DM | 4.87 |
| NDF, % of DM | 32.77 |
| ADF, % of DM | 17.35 |
| Lignin, % of DM | 4.67 |
| Ash, % of DM | 3.43 |
