# Supplementary material for: The Effects of Weaning Methods on Gut Microbiota Composition and Horse Physiology
Source: Front Physiol. 2017 Jul 25;8:535. doi: 10.3389/fphys.2017.00535 (PMC5524898; doi:10.3389/fphys.2017.00535)
Supplement: Table S12 — Link between the three gut community types defined at day 3 post-weaning, the gut fermentation parameters and the host parameters. [file Table12.DOCX]

**Table S12.** Link between the three gut community types defined at day 3 post-weaning, the gut fermentation parameters and the host parameters

|  | Treatment^1^ | | |  | |  |  | | |  |
| --- | --- | --- | --- | --- | --- | --- | --- | --- | --- | --- |
| Item | C1 | C2  MG  HG | | C3 | | SD | *P*-value^2^ | | |  |
| Body weight, Kg | 163 | 165 | | 159 | | 23.8 | 0.61 | | |  |
| Average daily gain, Kg | 0.46 | 0.45 | | 0.31 | | 0.29 | 0.01 | | |  |
| Feces pH | 6.64 | 6.64 | | 6.70 | | 0.21 | 0.28 | | |  |
| Total SCFA in feces, mM | 19.94 | 20.77 | | 17.26 | | 5.39 | 0.0009 | | |  |
| Individual SCFA in feces, mol/100 mol |  |  |  |  |  | | |  |  | |
| Acetate | 13.71 | 13.76 | | 11.75 | | 3.58 | 0.001 | | |  |
| Propionate | 3.65 | 3.78 | | 2.93 | | 1.23 | 0.0001 | | |  |
| Butyrate | 0.96 | 1.20 | | 0.91 | | 0.34 | 0.004 | | |  |
| Isobutyrate | 0.28 | 0.34 | | 0.30 | | 0.13 | 0.20 | | |  |
| Isovalerate | 0.29 | 0.38 | | 0.30 | | 0.13 | 0.009 | | |  |
| Valerate | 0.17 | 0.24 | | 0.16 | | 0.07 | 0.01 | | |  |
| Caproate | 0.08 | 0.04 | | 0.07 | | 0.05 | 0.05 | | |  |
| Isocaproate | 0.01 | 0.02 | | 0.01 | | 0.02 | 0.40 | | |  |
| Total iso | 0.75 | 0.98 | | 0.79 | | 0.28 | 0.01 | | |  |
| Acetate:propionate ratio | 3.87 | 3.92 | | 4.23 | | 0.68 | 0.02 | | |  |
| Individual taxa, log 10 g DNA in feces |  |  | |  | |  |  | | |  |
| Bacteria | 11.22 | 11.46 | | 11.25 | | 0.15 | 0.049 | | |  |
| Protozoa | 9.35 | 9.44 | | 9.34 | | 0.48 | 0.063 | | |  |
| Fungi | 9.24 | 8.18 | | 8.62 | | 0.74 | 0.0006 | | |  |
| Cortisol in saliva, μg/L | 0.40 | 0.48 | | 0.59 | | 0.18 | 0.004 | | |  |
| Telomere length relative to interferon-γ | 0.92 | 1.00 | | 0.99 | | 0.11 | 0.05 | | |  |
| Parasite egg counts/g feces | 694 | 619 | | 695 | | 684 | 0.89 | | |  |

^1^ C1=community type 1, C2= community type 2, C3= community type

^2^ Effect of gut community type
