# Supplementary figures and images for: The Effects of Weaning Methods on Gut Microbiota Composition and Horse Physiology
Source: Front Physiol. 2017 Jul 25;8:535. doi: 10.3389/fphys.2017.00535 (PMC5524898; doi:10.3389/fphys.2017.00535)

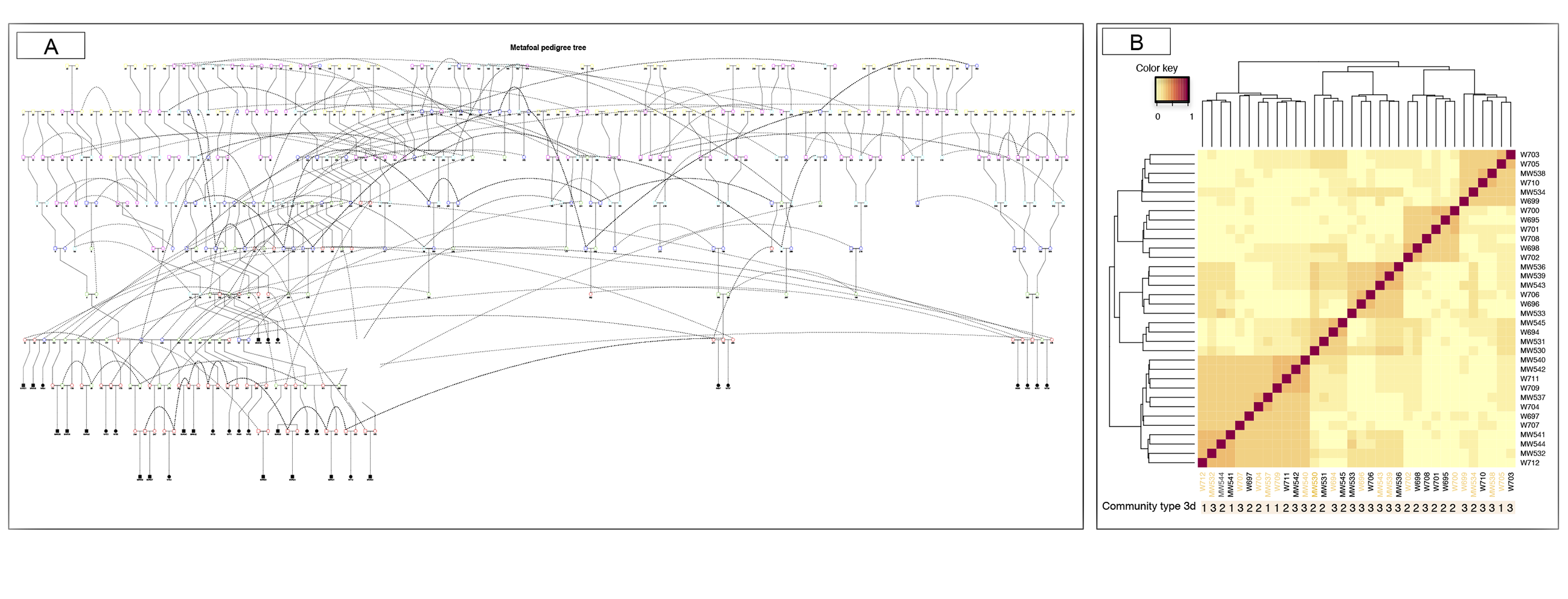

Supplement: Figure S1 — Genetic resemblances between the 34 foals in the experiment (A). Pedigree plot. A six-generation pedigree plot is illustrated, with different shapes for male (squares) and female (circles). The shapes are black for the 34 foals in the study. (B) Heatmap of the kinship coefficient matrix, which assess the genetic resemblance between individuals. Each entry in the matrix is the kinship coefficient between two subjects. Animals are arranged in the order of their genetic relatedness; genetically similar animals are near each other. Note that the diagonal elements did not have values above unity, showing no consanguineous mating in the families. Animals pertaining to progressive weaning are colored in orange. The community type at 3 days post-weaning is delineated next to the animal name. At 3 days post-weaning, the distribution of gut community types displayed between individuals with higher genetic relatedness was equal (1:1:1 of Community1: Community 2:Community3). However, 80% of the individuals from the community type 1 were siblings. [file Image1.tif]

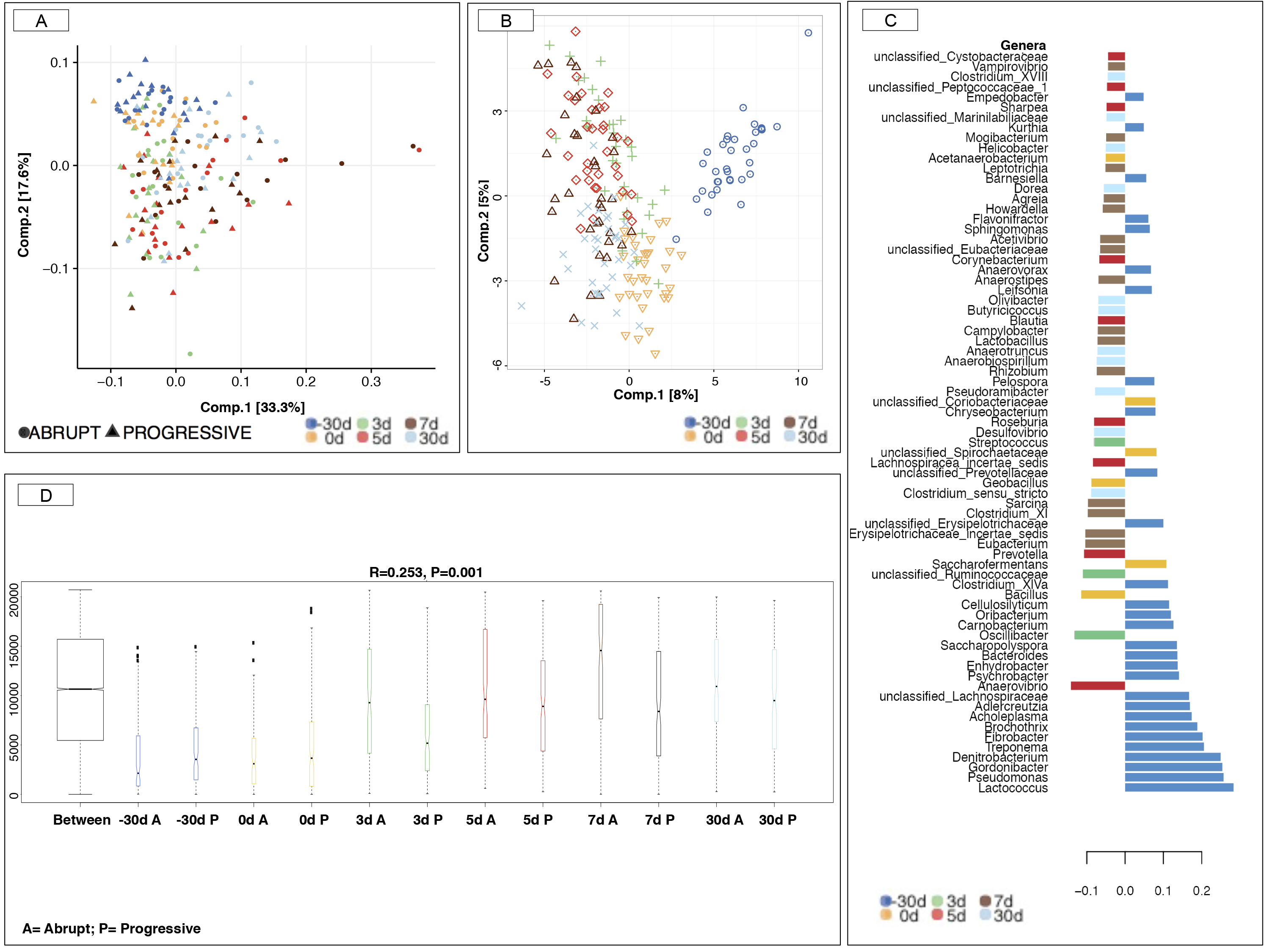

Supplement: Figure S2 — Multidimensional reduction methods for elucidating the effects of weaning method and time on gut microbiota composition. (A) Correspondence analysis of Unifrac distances to compare gut microbiota composition at the level of genus across time. Both axes 1 and 2 were plotted. Together they explained 50.9% of whole variation. The point's shape indicates the type of weaning (round: brutal; triangle: progressive); (B) sPLS-DA of gut microbiota composition at the level of genus across time; (C) Contribution plot of sPLS-DA model of each genera across time. The plot displays the importance of each genus in the sPLS-DA model and in which time point they are the most abundant (contrib = “max”), according to the median (method = “median”); (D) ANOSIM results to test for differences in gut microbiota composition at the level of genus between weaning methods across time. The analysis showed an R = 0.253 (P < 0.001) across time, indicating that all samples within a time point were more similar to each other than to any other samples from different time points. In all cases, −30 days is shown in blue, 0 days in orange, 3 days in green, 5 days in red, 7 days in brown and 30 days post-weaning in light blue color. [file Image2.TIF]

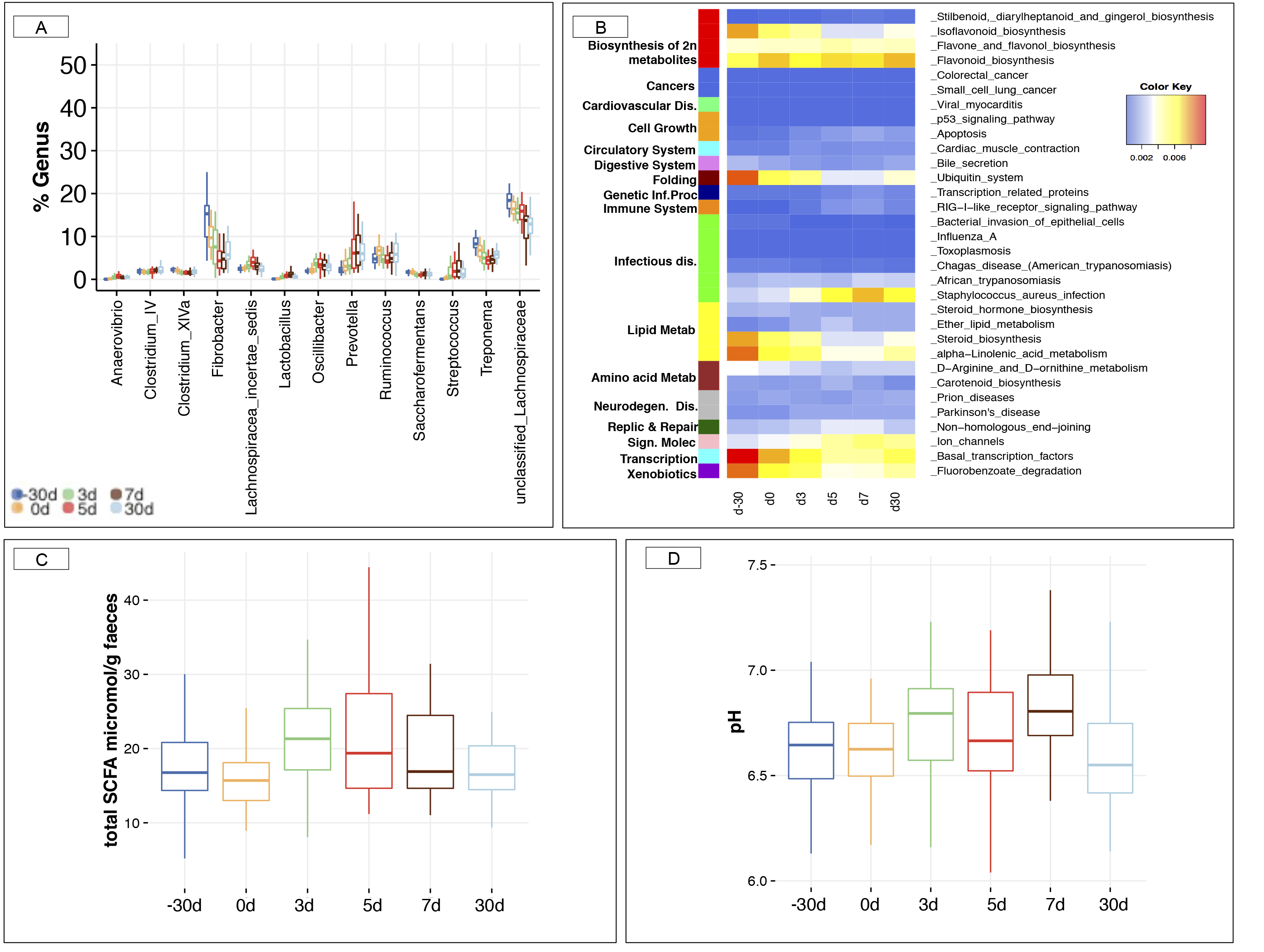

Supplement: Figure S3 — Genera and KEGG pathways abundance across time. (A) Boxplot representation of the most abundant genera that significantly shifted after maternal separation at weaning; (B) The KEGG pathways differentially expressed across time (q < 0.01) and identified by PICRUSt tool were displayed using a heatmap. The subpathways were also represented in the lateral bar using different colors; (C) Boxplot representation of total short chain fatty acids concentration (μmol/g feces) across time; (D) Boxplot representation of fecal pH across time. In all cases, −30 days is shown in blue, 0 days in orange, 3 days in green, 5 days in red, 7 days in brown and 30 days post-weaning in light blue color. [file Image3.TIF]

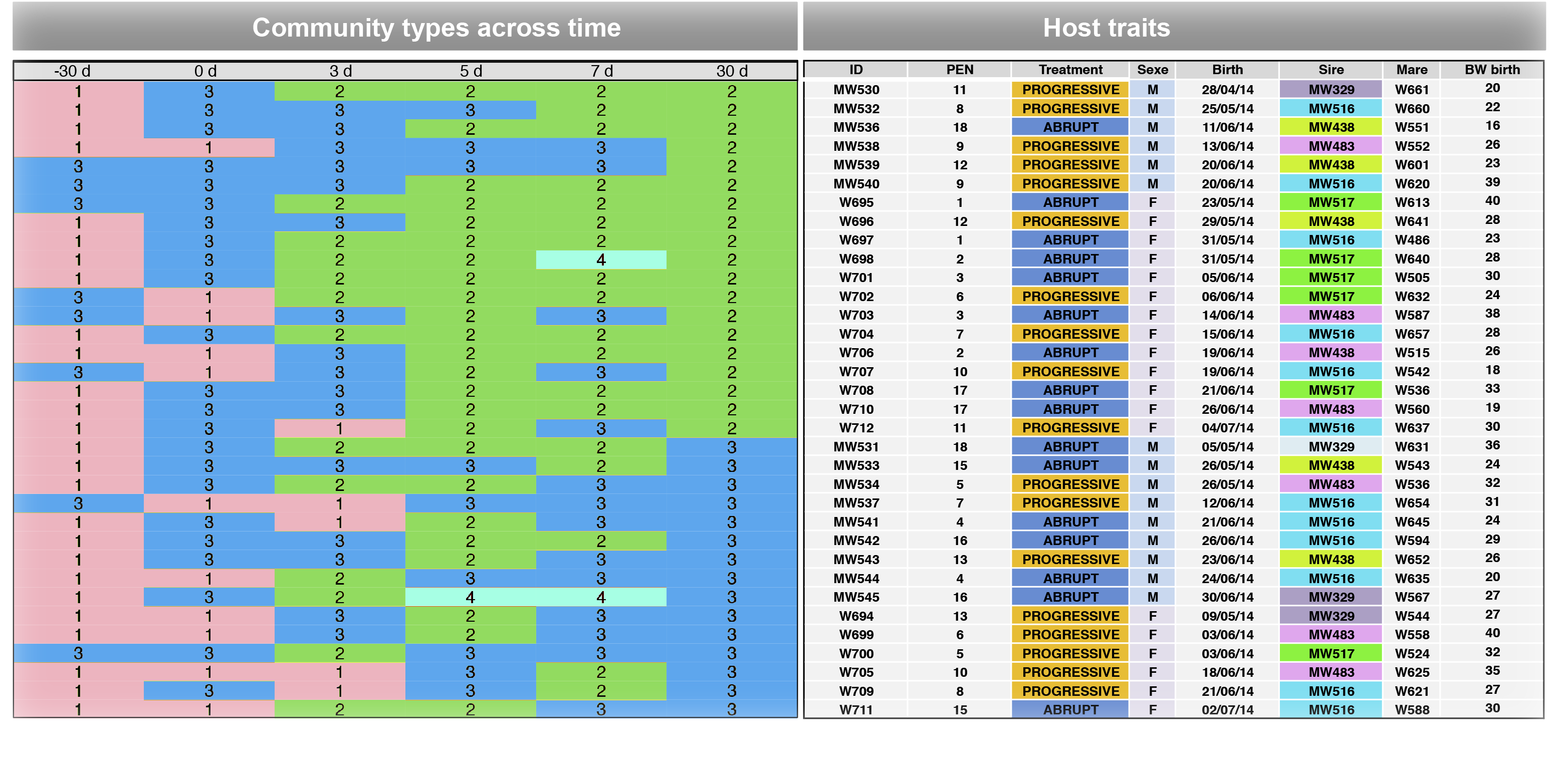

Supplement: Figure S4 — Dynamisms of the gut microbiota community types across time. In the cell plot, each column indicates one different time points, while each row displays the gut community type attributed to each animal. In all cases, pink color (community type 1), green color (community type 2), and blue color (community type 3). For each row in the cell plot, the information concerning the animal ID, weaning method, sex, sire, mare, and body weight at birth are depicted. [file Image4.TIF]

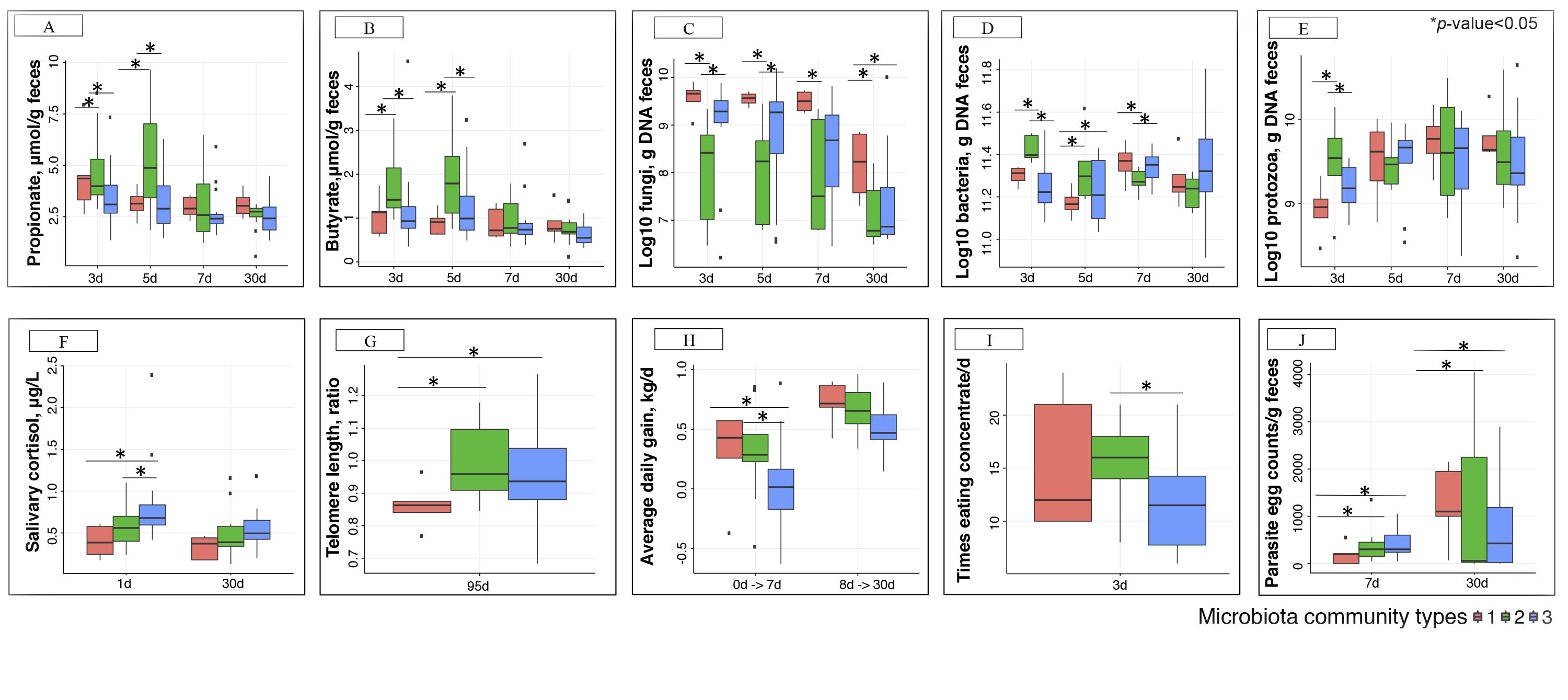

Supplement: Figure S5 — Effect of community type on performance and health parameters at 3 days post-weaning. (A) Boxplot representation of propionate (μmol/g feces) concentration between the three gut community types; (B) Boxplot representation of butyrate concentration (μmol/g feces) distribution between the three gut community types; (C) Boxplot representation of fungal loads (log 10 molecules, g of DNA in feces) between the three gut community types; (D) Boxplot representation of bacterial loads (log 10 molecules, g of DNA in feces) between the three gut community types; (E) Boxplot representation of protozoan loads (log 10 molecule, g of DNA in feces) between the three gut community types; (F) Boxplot representation of saliva cortisol concentration (μg/L) between the three gut community types; (G) Boxplot representation of the telomere length between the three gut community types relative to a control sample. The values reported in the plot correspond to the ΔΔCt between the Ct values of the amplified telomeric region and the Ct values of the single-copy gene (interferon-γ gene; IFGM) measured for each sample and relative to a control sample, in this case the value at day −73; (H) Boxplot representation of average daily gain (Kg/d) between the three gut community types; (I) Boxplot representation of frequency eating concentrate per day between the three gut community types; (J) Boxplot representation of parasite eggs counts (eggs/g feces) distribution across the three gut community types. In all cases, pink color represents the community type 1, green color represents the community type 2, and blue color represents the community type 3. *p < 0.05. [file Image5.TIF]
